# Identifiability, Sensitivity, and Genetic Algorithms in Bacterial Biofilm Selection Models

**DOI:** 10.1007/s11538-026-01693-5

**Published:** 2026-07-02

**Authors:** Stephen Williams, Daravuth Cheam, Michele K. Nishiguchi, Suzanne S. Sindi, Shilpa Khatri, Erica M. Rutter

**Affiliations:** 1 Department of Applied Mathematics, University of California, Merced, California US; 2 Department of Molecular and Cell Biology, University of California, Merced, California US; 3 Quantitative and Systems Biology, University of California, Merced, California US

**Keywords:** Parameter identifiability, Optimal experimental design

## Abstract

Bacteria often develop distinct phenotypes to adapt to environmental stress. In particular, they can produce biofilms, dense communities of bacteria that live in a complex extracellular matrix. While previous studies have investigated how bacterial biofilms are regulated under laboratory conditions, they have not considered (1) the data requirements necessary to estimate model parameters and (2) how bacteria respond to recurring stressors in their natural habitats. To address (1), we adapted a mechanistic population model to explore the dynamics of biofilm formation in the presence of predator stress, using synthetic data. We used a Maximum Likelihood Estimation framework to measure crucial parameters underpinning the biofilm formation dynamics. We used genetic algorithms to propose an optimal data collection schedule that minimised parameter identifiability confidence interval widths. Our sensitivity analysis revealed that, within the explored regimes, we could simplify the binding dynamics and eliminate biofilm detachment. To address (2), we proposed a structured version of our model to capture the long-term behaviour and evolutionary selection. In our extended model, the subpopulations feature different maximal rates of biofilm formation. We compared the selection under different predator types and amounts and identified key parameters that affected the speed of selection via sensitivity analysis.

**Supplementary Information:**

The online version contains supplementary material available at 10.1007/s11538-026-01693-5.

## Introduction

Many bacteria are cosmopolitan, but can survive under extreme conditions such as high or low temperature, humidity, salinity, and pH (Wang et al. 2015; Merino et al. 2019; Kochhar et al. 2022). Bacteria have evolved to employ several key strategies to maintain the flexibility needed to address such various environmental challenges (Coker 2023; Marzban and Tesei 2025; Somayaji et al. 2022). One such strategy is to adopt distinct ecotypes, observable differences between members of the same species in response to their environment. One type of adaptive response is the ability to form biofilms in response to both abiotic and biotic conditions.

Biofilms are communities of bacteria spread throughout an extracellular matrix, composed of secreted substances, particularly polysaccharides, proteins, and DNA (Flemming et al. 2016; Flemming and Wingender 2010). Biofilm formation can confer various forms of resilience to its occupants by creating spatially favourable distributions. This enables the bacteria to collectively share metabolic workload, and benefit from cooperative behaviours like quorum sensing (Zhang et al. 2024; Li and Tian 2012).

Free-living or planktonic bacteria move via diffusion or active swimming and readily adhere to surfaces or each other. These adhered bacteria become centres for more bacteria to attach, forming microcolonies that grow into mature biofilms through further attachment and internal replication. Researchers have examined the formation process using experiments (Merritt et al. 2006), numerical simulations using hydrodynamic theory (Zhao and Wang 2017), ordinary and partial differential equation models (Trubenová et al. 2022; D’Acunto et al. 2021), and agent-based modelling (Nagarajan et al. 2022). These studies can provide valuable insights for several applications, including the prevention of biofouling (Elumalai et al. 2024; Liu et al. 2024a; Flemming 2020), infections in medical devices (Høiby et al. 2010), and applications to bioremediation (Muhammad et al. 2020). By understanding biofilm formation dynamics under environmental stress, we also gain a crucial understanding of the wider ecological role of bacteria (Cohen et al. 2019, 2020; Hall-Stoodley et al. 2004).

Differential equation models, where dynamics reflect expected *in vivo* behaviours, offer one way to model biofilm formation dynamics (Bottomley and Eberl 2024; Garde et al. 2020). In our study, we use these models to gain a deeper understanding of biofilm formation, particularly under external stressors. Our study addresses the types and amounts of data required to parameterise such models, as well as how existing forms of models can be adapted to capture long-term effects that are maintained through selection. Initially, we will focus on the model presented in (Seiler et al. 2017), which utilises a set of differential equations to model a biofilm formation experiment under predation. The mathematical dynamics governing each population within such models follow established behaviours (Murray 2002). While these models are intuitively appealing due to their mechanistic interactions, they also make strong assumptions and introduce numerous potentially unknown parameters (Gunawardena 2014; Villaverde et al. 2023). As such, understanding how to measure these parameters and quantify their uncertainties is imperative (Kirk et al. 2013; Simpson et al. 2024). Therefore, we will determine a practical experimental setup that optimises the measurability of the parameters underlying our model. Additionally, this study enables us to explore how varying external stressors for such systems can lead to adaptations within the population through selection. The methodologies applied in this study allow us to develop a long-term model from a short-term model to study selection dynamics.

Our first aim is to evaluate this model using structural identifiability, practical identifiability, and sensitivity analysis, and use that information to propose an optimal data collection schedule (Banks et al. 2014). Assuming that the model parameters can be categorised into *a priori* known and unknown categories, we determine the values and associated uncertainties of the unknown parameters. We validate the inverse problem on synthetic datasets generated from a known baseline parameter set, a common approach in such studies (Bürger et al. 2023; Liu et al. 2024b). The resulting re-estimated parameters and their confidence intervals are calculated from the synthetic dataset. Using relative practical identifiability, we use a genetic algorithm to propose optimal data sampling schedules. Further, we employ sensitivity analysis to determine which parameters drove significant differences in population dynamics. Finally, informed by the sensitivity and identifiability analysis results, we propose a simpler model that produces similar dynamics.

Our second aim is to model bacterial adaptation in a controlled setting. Researchers often use long-term evolutionary experiments (LTEE) to study these phenomena (Lenski 2017, 2023). LTEE involves the culture of numerous, initially identical, bacterial populations. Periodically subcultured populations in the LTEE allow the bacteria to proliferate continuously, resulting in many generations. By exposing subsets of these isolated cultures to fixed stressors, their development is guided by those selection pressures. The bacteria, in turn, will evolve different dynamics to offset the stressors. In a differential equation model, adaptation across generations can be thought of as bacteria changing the values of their fixed model parameters, or through generic functions that interpolate between conditions (Lewkiewicz et al. 2022). Assuming our model’s inverse problem is well-posed, experimental data could be used to perform the inverse problem at different generations of the LTEE, and then measure the various parameter values. However, how these parameter values change in this scenario is spontaneous, and there is no measurement of how they transition from their initial to final values. Since the initial model lacks a mechanism that enables these long-term changes to occur continuously, we will introduce a structured variant of this model, where the bacterial community is comprised of individuals with intrinsic differences in their dynamics. We will demonstrate using a systematic resetting of the population, mirroring an LTEE, that selection is then possible for such a structured model.

Section 2 introduces the mathematical models and methods applied in this work. In Section 2.1, a short-term model is outlined, and in Section 2.2, we outline a modified long-term model. Section 2.3 through Section 2.8 introduce structural and practical identifiability, the genetic algorithms techniques, and the sensitivity analysis. Section 3 discusses our results. We first outline the results of the short-term model’s structural and practical identifiability, before proposing a genetic algorithm approach to improve the model’s identifiability. Next, we introduce the long-term model in Section 3.4, and detail the sensitivity analysis of this model in Section 3.5. Finally, a discussion of the results is given in Section 4.

## Models and Methods

In this section, we begin by introducing the models used to explore the dynamics of biofilm formation under predation. Following this, we outline the techniques used to simulate these dynamics and describe the tools used to analyse model outputs.

### Short-term Biofilm Formation Model

**Fig. 1:**
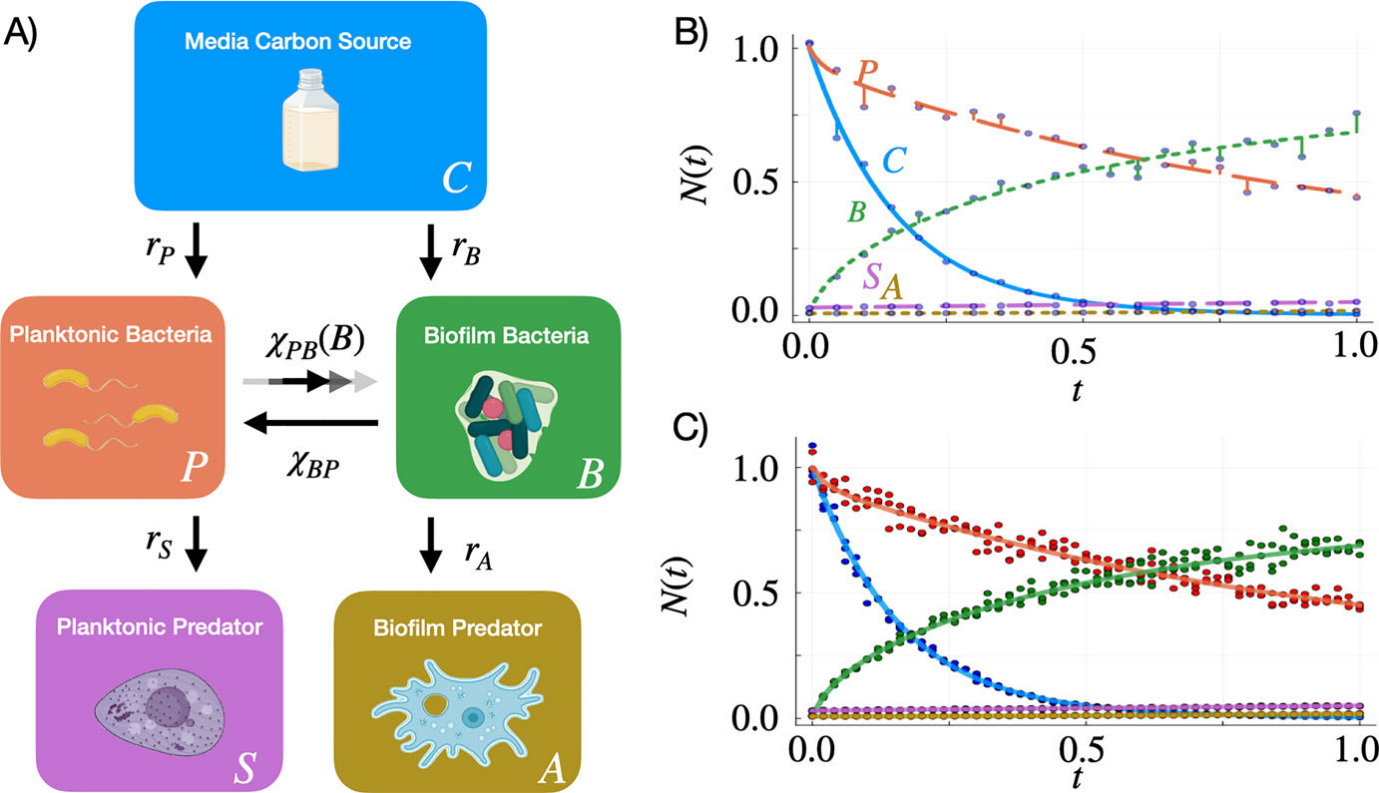
**Model schematics and example solutions.** (A) Schematic of the short-term ODE system (Equation (1)-Equation (5)). Both bacterial phenotypes consume the media carbon source, *C*, to reproduce, with growth rates $r_P$ for the planktonic cells *P* and $r_B$ for the biofilm cells *B*. Bacteria transition from a planktonic to a biofilm phenotype at a rate dependent on biofilm size, $\chi _{PB}(B)$. The biofilm bacteria detach, becoming planktonic at a constant rate $\chi _{BP}$. The bacteria are consumed by their associated predators. (B) An example solution (solid, dashed, and dotted lines) with initial conditions $[C_0, P_0, B_0, S_0, A_0] = [1.0, 1.0, 0.01, 0.03, 0.008]$ and an example synthetic dataset (circular purple points). Here, *N*(*t*) is the population size of each compartment in the model at time *t*. Vertical lines indicate the distance between the synthetic data and the noise-free solutions. Colours correspond to the compartments in A. (C) The best-fit solution (solid lines) compared to moderately noisy synthetic data (scatter points, introduced in Section 2.3) for the system in (B). *N*(*t*) again shows the population size of each compartment. The synthetic dataset consists of three biological replicates, each with 51 uniformly spaced time points (color figure online)

In the first part of our study, we focus on the dynamics of a population within one self-contained experimental setting. To represent the flow of nutrients through our biofilm-forming system, we use a stoichiometric mathematical model proposed in (Seiler et al. 2017), see Figure 1A. The different populations are measured using state variables, which indicate the carbon content contained in these populations, access to which is limited by the initial content present (Bren et al. 2013; Hornung et al. 2018). Our equations, which model the state variables, are given by

1 $$\begin{aligned} \frac{dC}{dt}&= - \underbrace{r_P \frac{C}{C+H_C} P}_{\begin{array}{c} \text {Carbon consumed}\\ \text {by Planktonic} \end{array}} - \underbrace{r_B \frac{C}{C+H_C} B}_{\begin{array}{c} \text {Carbon consumed}\\ \text {by Biofilm} \end{array}}, \end{aligned}$$

2 $$\begin{aligned} \frac{dP}{dt}&= \underbrace{e_b r_P \frac{C}{C+H_C} P}_{\begin{array}{c} \text {Planktonic growth} \end{array}} - \underbrace{r_S\frac{P}{P+H_P}S}_{\begin{array}{c} \text {Planktonic consumed}\\ \text {by predator} \end{array}} - \underbrace{\frac{a\chi _{PB}^{\max } + B\chi _{PB}^{\min }}{a + B}P}_{\begin{array}{c} \text {Planktonic attaching}\\ \text {to Biofilm} \end{array}} + \underbrace{\chi _{BP}B}_{\begin{array}{c} \text {Biofilm}\\ \text {Detachment} \end{array}} , \end{aligned}$$

3 $$\begin{aligned} \frac{dB}{dt}&= \underbrace{e_b r_B \frac{C}{C+H_C} B}_{\begin{array}{c} \text {Biofilm growth} \end{array}} - \hspace{1.5mm} \underbrace{r_A\frac{B}{B+H_B}A}_{\begin{array}{c} \text {Biofilm consumed}\\ \text {by predator} \end{array}} \hspace{2mm}+ \underbrace{\frac{a\chi _{PB}^{\max } + B\chi _{PB}^{\min }}{a + B}P}_{\begin{array}{c} \text {Planktonic attaching}\\ \text {to Biofilm} \end{array}} - \underbrace{\chi _{BP}B}_{\begin{array}{c} \text {Biofilm}\\ \text {Detachment} \end{array}} , \end{aligned}$$

4 $$\begin{aligned} \frac{dS}{dt}&= \underbrace{e_S r_S\frac{P}{P+H_S}S}_{\begin{array}{c} \text {Planktonic predator}\\ \text {growth} \end{array}} , \end{aligned}$$

5 $$\begin{aligned} \frac{dA}{dt}&= \underbrace{e_A r_A\frac{B}{B+H_A}A}_{\begin{array}{c} \text {Biofilm predator}\\ \text {growth} \end{array}}. \end{aligned}$$

Here, the state variables represent the compartments present in the system: the media source (*C*), planktonic (e.g., free-swimming) bacteria (*P*), biofilm bacteria (*B*), planktonic predator (*S*), and biofilm predator (*A*). The carbon source (Equation (1)) is consumed by both planktonic and biofilm cells. Equation (2) and Equation (3) represent planktonic and biofilm bacteria, respectively. Their similar structure includes (a) carbon-limited growth, (b) predation by their respective predators, (c) biofilm attachment, and (d) biofilm detachment. The attachment of planktonic bacteria to biofilm depends on the size of the biofilm: the bacteria attach at a maximum rate $\chi _{PB}^{\max }$ for small biofilm population sizes, in the limit of large biofilms, the rate tends toward $\chi _{PB}^{\min }$. The parameter *a* is a fixed biofilm size, acting as a transition point, with the attachment rate smoothly transitioning between these maximum and minimum values as the biofilm grows in size beyond *a*. The detachment rate is proportional to the size of the biofilm, with a constant rate $\chi _{BP}$. Equation (4) and Equation (5) represent the planktonic and biofilm predators, which grow from consuming their respective food sources. In Equation (1)-Equation (5), all growth and consumption functions follow a type-II functional response (Gesztelyi et al. 2012). A schematic of nutrient flow is given in Figure 1A. An example solution is shown in Figure 1B (solid line) along with corresponding synthetic data, with Figure 1C showing a fit from re-estimated parameters.

Supplementary Table 1 presents the nominal values of the parameters used throughout our results. These values were calculated by non-dimensionalising the values from (Seiler et al. 2017) (see Supplementary Section 1). The non-dimensionalisation was chosen to provide consistency when comparing the relative sizes of the populations, regardless of whether they are in liquid (media, planktonic, or planktonic predator) or on the surface (biofilm, biofilm predator). However, our non-dimensional parameters implicitly depend on the geometry of the container used in experiments; thus, they must be re-determined for other experimental setups. Throughout, when referencing the values in Supplementary Table 1, we will use a tilde (e.g., the nominal value of $\chi _{BP}$ will be denoted as $\tilde{\chi }_{BP}$). Our primary focus is to use this model to investigate changes in biofilm formation. Thus, we fix all parameters in the model except those most relevant to biofilm formation: $\chi _{\max }, \chi _{\min }, a,$ and $\chi _{BP}$. For a given synthetic dataset, our analysis will focus on re-estimating the values of these parameters and determining their uncertainties.

### Long-term Structured Biofilm Model

The parameters in the model of Sect. 2.1 are fixed constants, which means that the bacteria cannot adopt different dynamics or adapt. Previous experimental literature has demonstrated that protozoan predation selects for defensive traits in bacteria, specifically regarding the formation of biofilms (e.g., microcolony formation) (Matz and Kjelleberg 2005). Therefore, we wish to model and understand the long-term evolution of the ability of bacteria to form biofilms under generations of predation. To study the long-term evolutionary dynamics of bacterial phenotypes, we propose a parameter-structure population model. In this updated model, we assume that instead of having a single population of bacteria (and thus a single value of the constant parameter describing the binding dynamics), we have a collection of similar but slightly different subpopulations. These subpopulations will be represented using a large number of compartments, each with a different biofilm formation-associated trait (i.e., different parameter values). Within these populations, the value of their maximum binding rate, referred to in the short-term model as $\chi _{PB}^{\max }$, approximates a continuum of binding rates by taking values on the fine grid for each compartment. The modified set of equations governing the system is:

6 $$\begin{aligned} \frac{dC}{dt}&= - r_P \frac{C}{C + H_C}\mathcal {P} - r_B\frac{C}{C + H_C}\mathcal {B}, \end{aligned}$$

7 $$\begin{aligned} \frac{dP_i}{dt}&= e_b r_P \frac{C}{C + H_C} P_i - r_S \frac{\mathcal {P}}{\mathcal {P} + H_P}\frac{P_i}{\mathcal {P}}S - \frac{a}{a+\mathcal {B}} \chi _i P_i, ~~~ i\in (1,2, ..., N_c), \end{aligned}$$

8 $$\begin{aligned} \frac{dB_i}{dt}&= e_b r_B \frac{C}{C + H_C} B_i - r_A \frac{\mathcal {B}}{\mathcal {B} + H_B}\frac{B_i}{\mathcal {B}}A + \frac{a}{a+\mathcal {B}} \chi _i P_i, ~~~ i\in (1,2, ..., N_c), \end{aligned}$$

9 $$\begin{aligned} \frac{dS}{dt}&= e_S r_S \frac{\mathcal {P}}{\mathcal {P} + H_P}S, \end{aligned}$$

10 $$\begin{aligned} \frac{dA}{dt}&= e_A r_A \frac{\mathcal {B}}{\mathcal {B} + H_B}A, \end{aligned}$$

11 $$\begin{aligned} \mathcal {P}&= \sum _i P_i, \end{aligned}$$

12 $$\begin{aligned} \mathcal {B}&= \sum _i B_i. \end{aligned}$$

This set of equations is similar to Equations (1)-(5), with a few key differences: (1) the introduction of subpopulations within *P* and *B*, (2) the biofilm detachment rate, $\chi _{BP}$, is now assumed to be 0, and (3) the biofilm attachment rate has been simplified. Equations (7) and (8) describe the dynamics of the bacteria occupying the *i*th compartment. The total amount of planktonic and biofilm populations is the sum of the individual compartments (Equation (11) and Equation (12)). As before, there is growth from media source consumption and elimination due to predation. The growth and death terms are scaled to be proportional to the size of the compartment. The remaining term concerns the biofilm attachment rate, which remains dependent on the size of the underlying biofilm. However, the minimum attachment rate tends toward zero in the limit of large biofilms. Each of the $N_C$ ordered compartments has an associated biofilm maximum attachment rate, $\chi _i$, at a uniformly spaced set of values (i.e., $\chi _1< \chi _2 = \chi _1 + \Delta \chi< ... < \chi _N = \chi _1 + (N-1)\Delta \chi$). The bacteria are assumed to retain their maximum attachment rate $\chi _i$ upon transitioning between the phenotypes; thus, $P_i$ and $B_i$ are coupled, but do not interact with the other compartments. For clarity, we use the notation $\chi _{\max }$ in figures and discussions to represent the maximum value of the compartment. We select our range of $\chi _i$ so that evolving populations never reach the endpoints $\chi _1$ or $\chi _N$ (Rutter et al. 2017). To approximate a continuum of $\chi _{\max }$ values, we used $N_c=2000$ compartments in our results (the convergence study motivating this choice is presented in Supplementary Section 5). The dynamics for the carbon media source (Equation (6)) and both predators (Equation (9) and Equation (10)) are analogous to the previous model.

To solve Equations (6)-(12), the initial conditions are the same for *C*, *S*, and *A*, but the initial condition for $P_i$ or $B_i$ must be a distribution. To solve our system, we take the initial distribution:

13 $$\begin{aligned} P_i = \frac{\gamma }{\sigma _\chi } \exp \left( -\frac{\left( i-i_c\right) ^2}{2\sigma _\chi ^2} \right) , \end{aligned}$$

where $\gamma$ is a constant chosen so that the sum across all compartments $\sum _i^{N_c}P_i=1$. The distribution has some width, measured by the standard deviation $\sigma _\chi$, and a mean $i_c$ corresponding to a binding rate of $\chi _{i_c}$. We start the experiment with no biofilm present, $B_i=0$.

To mimic the LTEE setup, the structured model is repeatedly solved to $t=1$, representing one generation, and re-seeded. This choice is motivated by an envisioned LTEE experimental setup in which the supernatant (containing media, planktonic predators, and biofilm predators) is removed from the system. Following this, the preserved biofilm is mechanically broken up to become the new planktonic population (e.g., initial conditions $P_i = B_i^\text {old}/\sum _i B_i^\text {old}$ and $B_i^\text {new} = 0.0$). New media and an appropriate level of predators are then reintroduced (e.g., *C*, *S*, and *A* are reset to their initial values). By iterating this process, simulating the system to $t=1$ and then resetting the system using only the biofilm population, selection is enabled across multiple generations.

### Model Numerical Solutions and Synthetic Data Construction

The mathematical models of the populations (see Section 2.1 and Section 2.2) are solved numerically using the Julia package DifferentialEquations (Rackauckas and Nie 2017), specifically the Tsit5 implementation of the Runge-Kutta 4(5) (Tsitouras 2011).

We utilise noisy synthetic datasets to assess our ability to measure model parameters at various levels of sparsity. To generate our synthetic data for compartment *i*, $Y_i(t)$, we use the following statistical model:

14 $$\begin{aligned} Y_i(t) = X_i(t) \mathcal {E}_i, ~~~~~\text {where} ~~~~~ \mathcal {E}_i \sim \text {Log-Normal}(\mu ,\sigma ). \end{aligned}$$

For each data point $X_i(t)$, we modify the exact numerical solution according to multiplicative noise drawn from a Log-normal distribution (Murphy et al. 2024). A synthetic dataset example is overlaid on the numerical solution in Figure 1B. We select the log-normal distribution to preserve the positivity of the synthetic data. The value of our Log-normal is given by $\mathcal {E}_i = \exp (\mu _l + \sigma _l \epsilon _i)$, where $\mu _l$ and $\sigma _l$ are some real numbers and $\epsilon _i$ is a standard normally distributed random variable (i.e., mean zero and standard deviation one). These values $\mu _l$ and $\sigma _l$ tell us the mean and standard deviation of $\log (\mathcal {E}_i)$, not of $\mathcal {E}_i$. We represent our distribution’s mean and standard deviation as $\mu$ and $\sigma$, respectively. The relationship between these values is as follows,

15 $$\begin{aligned} \mu _l = \log \left( \frac{\mu ^2}{\sqrt{\mu ^2 + \sigma ^2}}\right) , \end{aligned}$$

16 $$\begin{aligned} \sigma _l^2 = \log \left( 1 + \frac{\sigma ^2}{\mu ^2}\right) . \end{aligned}$$

Using the Julia packages Distributions and Random, the values $\mu _l$ and $\sigma _l$ were used to generate samples from the Log-normal distributions (Besançon et al. 2021). We assume that such multiplicative noise is appropriate to capture the errors present in measurements of each compartment, and that the values of $\mu$ and $\sigma$ are the same for each compartment. Throughout all synthetic data, we use the value of $\mu =1$. While we assume we know the value of $\sigma$ *a priori*, it can easily be estimated by extending our parameter estimation approach, using $\sigma$ as an additional parameter to be estimated, if it’s not known in advance, as presented in (Simpson et al. 2020).

In experiments, the measurement of bacterial population size through optical density or Colony-Forming Units is often a balance between speed and accuracy. Modern techniques report coefficients of variation in the range of 1-5% (corresponding to values as low as $\sigma =1\times 10^{-2}$) (Rahman and Butzin 2024; Martini et al. 2024). In Section 3.1, we use an artificial value of $\sigma = 2.5 \times 10^{-3}$ to compare the relative difference between parameter estimation bounds, particularly in cases with very limited datasets. This noise represents an absolute best-case scenario value for these parameter estimate confidence bounds. At this noise level, we observe a range of practical identifiability across our parameters of interest, with some parameters estimated with very narrow 95% confidence intervals, while others have wide intervals.

### Model Structural Identifiability

The first step in model evaluation is determining if the system is structurally identifiable. This intrinsic model property determines whether, given its noise-free outputs, the parameters within the model can be re-determined uniquely (Villaverde et al. 2023). To determine whether the models presented are structurally identifiable, we used the online Maplecloud Structural Identifiability Toolbox (Hong et al. 2020). This program uses the Structural Identifiability Analyser (SIAN) (Hong et al. 2019). We determined whether model parameters were globally identifiable by providing different information availability. For example, in real-world experiments, the concentration of planktonic bacteria, the concentration in the biofilm, and the number of predators are relatively straightforward to measure (using Colony Forming Unit measurements (CFU), optical density (OD), haemocytometry, etc.) (Pan et al. 2014; Beal et al. 2020; Zhang et al. 2020). However, measuring the concentration of nutrients in the media, which can be done using techniques like liquid chromatography mass spectrometry, is comparatively impractical or destructive (Floris et al. 2019). Restricting access to information about the media in a dataset (i.e., omitting synthetic data regarding *C* throughout simulations) may pose issues regarding what parameters can be determined. Thus, structural identifiability analysis allows us to determine whether information about parameters within the system is even possible.

### Model Practical Identifiability

Throughout our study, we will assess whether the model is practically identifiable for given data scenarios . This analysis allows us to estimate the parameters within the model and quantify these estimates with appropriate statistical evidence .

We use the Maximum Likelihood Estimator (MLE) for a given set of synthetic data to re-estimate the baseline parameters used to generate the synthetic data (Simpson and Baker 2025). To do this, suppose we have data $Y_{i,j}(t_k)$ at a specific time $t_k$, where *i* represents the state variable and *j* represents the biological replicate. For a given initial guess at the baseline parameters $\boldsymbol{\theta }$, we can use our mathematical model to calculate what the parameter guess predicts the state variables should be, $X_i(t_k|\boldsymbol{\theta })$. The probability of the measurement $Y_{i,j}(t)$, $p(Y_{i,j}(t_k)/X_i(t_k;\boldsymbol{\theta }) ~|~ \text {Log-normal}(\mu ,\sigma ))$, can be calculated since the distribution of the synthetic data is known. Working with the log-probability allows us to write the log-probability of the whole dataset as

17 $$\begin{aligned} \ell = \sum _{i,j,k} \log \left[ p\left( \frac{Y_{i,j}(t_k)}{X_i(t_k;\boldsymbol{\theta })}\right) \right] . \end{aligned}$$

We have dropped the explicit error distribution label inside $p(\cdot )$ for simplicity. The best parameter set $\boldsymbol{\theta }_{opt}$ is the one which maximises the value of $\ell$ (equivalently, it is the parameter set which maximises the probability of measuring the data under our statistical model). We will refer to the optimal value as $\ell _p$. Hence,

18 $$\begin{aligned} \boldsymbol{\theta }_{opt} = \mathop {\mathrm {arg\,max}}\limits _{\boldsymbol{\theta }} \sum _{i,j,k} \log \left[ p\left( \frac{Y_{i,j}(t_k)}{X_i(t_k;\boldsymbol{\theta })}\right) \right] . \end{aligned}$$

To calculate this, we used the NLopt Julia package (Johnson 2007). Using an initial guess of the values of the parameters, the Nelder-Mead algorithm allows the optimal parameter values of Equation (18) to be found (Galántai 2024). Throughout our results, we use a single initial guess. A discussion of this choice is presented in Supplementary Section 3, where the initial guess is found to have little impact on the parameter estimation.

Without proper context, these estimated values can be somewhat misleading. The model is not practically identifiable with a given dataset if two distinct parameter sets provide the same log likelihood values. To quantify whether our parameters are practically identifiable, we calculated profile likelihood by fixing one parameter of interest at a time and then re-estimating the remaining parameters (Raue et al. 2009; Simpson and Baker 2025). For example, suppose we have measured the optimal values for our dataset $\boldsymbol{\theta }_{opt} = (\bar{\theta }_1,\bar{\theta }_2)$. We can, for some small value $\delta$, construct a new parameter set $\boldsymbol{\theta }' = ((1+\delta )\bar{\theta }_1, \theta _2)$. Where $(1+\delta )\bar{\theta }_1$ is fixed, and $\theta _2$ is re-estimated to maximise the log likelihood of our dataset. In that case, the log likelihood $\ell _p(\delta )$ must necessarily be less than the true optimal $\ell _p$ if $|\delta |>0$. By examining the value of $\bar{\ell }_p = \ell _p - \ell _p(\delta )$ across a range of values of $\delta$, we can determine how sensitive the estimation is to univariate variation. $\bar{\ell }_p$ is $\chi ^2$-distributed (Li and Babu 2019) and, therefore, we can determine a one-degree-of-freedom quantile (for our univariate parameter) above which the probability of observing the data is equal to or greater than a desired level. This quantile can be implemented using the Distributions Julia package and has a value $\ell _{crit} = -1.921$ for a 95% confidence level (Simpson and Baker 2025). A confidence interval width can be constructed by interpolating $\bar{\ell }_p - \ell _{crit}$ and finding its roots. We refer to this interval as the confidence interval width $CIW_{95\%}$. The function CubicSplineInterpolation from the Interpolations Julia package, alongside the Roots package’s function find_roots, were used to perform these calculations (Kittisopikul et al. 2025; Verzani 2020).

### Genetic Algorithm

In our log-likelihood parameter estimation in Sect. 2.5, Equation (17), no pre-supposition is made on the sampling times $t_k$. One obvious schedule choice is uniform; however, different parameters can drive dynamics at different population sizes. For example, as in Figure 1B, when there is a tiny biofilm population, the attachment rate $\chi _{PB}^{\max }$ dominates the biofilm population size changes. In contrast, $\chi _{PB}^{\min }$ barely has any impact. To measure $\chi _{PB}^{\max }$ as accurately as possible, it is better to have more data samples when the parameter is more impactful. We used a genetic algorithm to systematically construct schedules that allow us to measure a given parameter most effectively (Lam et al. 2024).

As described in Sect. 2.5, for a given synthetic dataset, a 95% confidence interval can be calculated for the best fit parameters. The genetic algorithm we employed closely follows previous work (Lam et al. 2024), but is explained here for clarity. The genetic algorithm first generates $M=100$ random schedules to determine the best possible sampling schedule. These schedules each consist of a fixed number of random timepoints; we generated 11, 21, and 51 sample point schedules in our case. The sample time points are generated by sampling a uniform distribution on (0, 1], with the initial condition always provided at t=0. Each new sample is initially checked to be greater than $\Delta t=0.01$ (14.4 minutes) away from other sample times. For each schedule, a synthetic dataset is generated, and the value of $CIW_{95\%}$ is found. The schedules can then be ranked according to the value of $CIW_{95\%}$, with the smallest $CIW_{95\%}$ values ranked highest. For the *M* cases, we use a cloning process to generate a new set of schedules (Lam et al. 2024). The best schedule is preserved unchanged. The new *i*th schedule in the cloned set is a copy of the $\text {ceil}(i/14)^2$th schedule in the previous set. This cloning method keeps many copies of the most highly ranked schedules and fewer, less highly ranked ones, up to the 51st best. Each cloned schedule time point is then modified by a value drawn from a normal distribution with a standard deviation $\sigma = 9\Delta t/N_{epochs}$. The values were constrained to remain in the range $(\Delta t,1-\Delta t)$. Because of the relatively large standard deviation of the cloning noise, points could move freely within this interval, exchange positions, and the minimum distance between scheduled points was not enforced from one epoch to the next. In later examples, three epochs were used to generate the optimal schedules, as in our results, relatively rapid convergence of optimal time points motivated this approach. (See points in epochs 2-5 in Figure 3B)

### Short-term Model Metrics and Sensitivity

We perform sensitivity analysis to assess the relationship between variations in biofilm dynamics parameters and their resulting population. Since we are interested in the dynamics of biofilm formation, we chose the final biofilm size as our quantity of interest, which we will refer to as $B^*$. We used Saltelli Sampling to generate a collection of 40,000 parameter sets, each of which the value of the parameters $\chi _{PB}^{\max },~\chi _{PB}^{\min },~a,~\chi _{BP}$ are varied over the range 10% to 190% of their nominal values (given in Supplementary Table 1) (Saltelli 2002). In our results, we scale the parameters to their nominal values to enable the best comparison between parameters, as well as to demonstrate that the MLE correctly estimates the parameters used to generate the synthetic data.

We use a variety of metrics to analyse the relationship between the parameters and $B^*$. To display the general trend between parameters and $B^*$, we present univariate scatter plots and their best-fit lines calculated via Julia’s CurveFit package. To measure linear relationships between the parameters and $B^*$, we calculate the Pearson and Partial correlations, the latter of which controls for the variation of other parameters. We also measure Spearman and Partial Rank Correlations, which allow us to remove the effects driven by outliers and measure the presence of monotonic relationships across the entire dataset. For a more in-depth discussion of these metrics, see (Marino et al. 2008). The above metrics were calculated using the Julia package GlobalSensitivity (Dixit and Rackauckas 2022).

Finally, we use Sobol Sensitivity indices to decompose the variance in the full dataset (Sobol 2001). Sobol indices examine the magnitude of the present variance, even if the relationships between parameters are non-linear. Additionally, Sobol sensitivity provides a method for examining the collective effects of parameters, assessing potential relationships between them by measuring second-order indices.

### Long-term Model Metrics and Sensitivity

In the long-term adaptation model, we apply sensitivity analysis methods to examine the relationship between model parameters and the change in biofilm binding rate. The average binding value can be found using

19 $$\begin{aligned} \bar{\chi }_{\max } = \frac{1}{\mathcal {B}} \sum _j\chi _j B_j. \end{aligned}$$

Applying Equation (19) to the initial condition for the biofilm, we can get the initial biofilm average binding $\bar{\chi }_{\max }^i$ (which corresponds to $\chi _{i_C}$ from Section 2.2). We then recalculate the average binding rate after 50 generations, $\bar{\chi }_{\max }^f$. Our quantity of interest is the ratio of these rates $\bar{\chi }_{\max }^f/\bar{\chi }_{\max }^i$, which measures the relative impact of selection in the population. The parameters examined in the sensitivity analysis are the amount of between generation resetting predators $S_0$ and $A_0$, the initial average binding rate $\chi _{i_c}$, and the variability of binding rates in the initial condition $\sigma _\chi$.

Sobol sensitivity was performed for a Saltelli sample using these methods , with 40,960 samples. The samples in this analysis were generated using the Python Library SALib sample.sobol’s function sample. The simulations were carried out in parallel using MATLAB to optimise the code runtime, the function ODE45 replacing the DifferentialEquations Tsit5 algorithm (Inc 2025). Since each sample’s execution took a relatively similar time, splitting the workload evenly between a MATLAB parallel pool of ‘threads’ workers heuristically resulted in the fastest execution. We saved the outputs for each parameter set, then analysed them separately using the Python Library SALib (Iwanaga et al. 2022). This library offers convenient functionality for importing parameters and datasets (note that results in Julia’s GlobalSensitivity must be generated locally).

## Results

Our results fall into two main sections. In Sects. 3.1 to 3.3, we will explore the short-term model presented in Sect. 2.1. We aim to determine whether the model parameters can be defined under specific data availability scenarios and assess the uncertainty in these measurements before examining the model’s sensitivity to variations in these parameters. In doing so, we show differences between the identifiability of the parameters and demonstrate that sampling schedule and frequency have a substantial effect on the parameters’ confidence intervals. Next, in Sections 3.4 to 3.5, we propose a new model for our system. This new model captures the impacts of selection on populations by introducing population structure within the bacterial compartments. Finally, we will measure this impact under specific conditions through a sensitivity analysis, showing that variability in bacterial parameters and predation type gives the most significant changes in the selection dynamics.

**Fig. 2:**
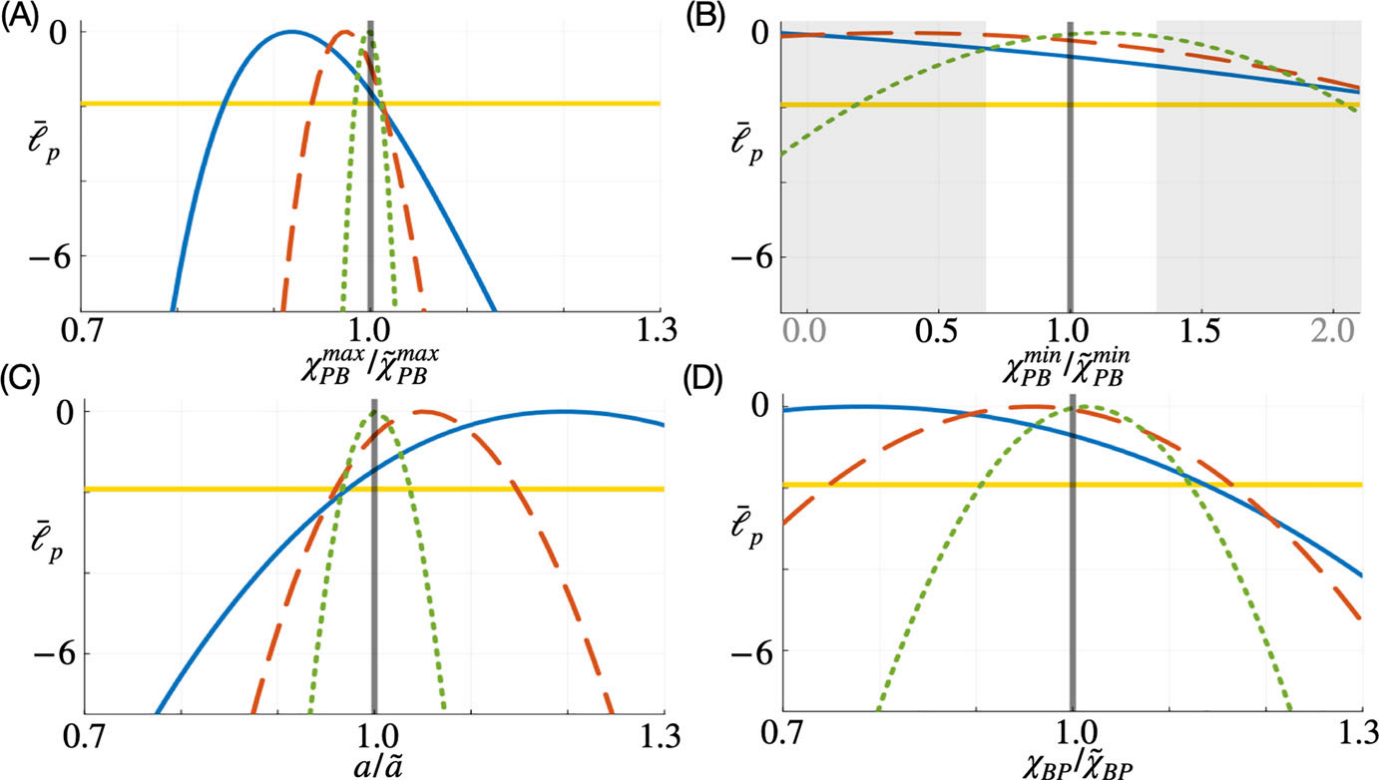
**Measuring practical identifiability using univariate profile likelihoods.** Log likelihoods relative to optimal solution $\bar{\ell _p}$ of parameters $\chi _{PB}^{max}$ (A),$\chi _{PB}^{min}$ (B), *a* (C), and $\chi _{BP}$ (D). In each case, the *x*-axis is normalised by the parameter’s nominal value (see Supplementary Table 1) and the grey vertical line represents the true value. For each parameter, the value at uniformly sampled time points of 11, 21, and 51 is given by the blue (solid), red (dashed), and green (dotted) curves, respectively. Intersections with the yellow curve correspond to 95% confidence intervals for the parameter values given the data (see Table 2 for the confidence interval width values). To highlight the difficulty in calculating the confidence interval in (B), vertical lines are given at $\theta /\tilde{\theta } = 0.7$ and $\theta /\tilde{\theta } = 1.3$, the x-axis range over which (A), (C), and (D) are presented (color figure online)

### Measuring the Impacts of Data Sampling Frequency on Model Practical Identifiability

Our first aim is to explore the structural and practical identifiability of the short-term model described in Sect. 2.1. To determine the global structural identifiability of our initial mathematical model (Equation (1)-Equation (5)), we used the SIAN toolbox (see Sect. 2.4 for details) (Hong et al. 2019). When information on the state variables [*C*, *P*, *B*, *S*, *A*] is available, the complete set of parameters and initial conditions present in Supplementary Table 1 is globally identifiable. In general, we will focus on the identifiability of the parameters $\chi _{PB}^{\max }, \chi _{PB}^{\min }, a,$ and $\chi _{BP}$, since these parameters determine the dynamics through which the bacteria transition between planktonic and biofilm phenotypes. We assume all other parameters are known exactly *a priori*. When limiting our interest to this subset of parameters, only the biofilm *B* needs to be measured for global identifiability.

To provide the best-case scenario for measuring these parameters practically, we will assume that we have access to measurements of all state variables. We generate a synthetic dataset with three biological replicates (described in Section 2.3) based on a uniform sample schedule as a proxy for experimental measurements. Additionally, the samplings include a measurement of the system’s initial condition. Figure 1B displays a synthetic data example using the nominal values from Supplementary Table 1 for a single biological replicate with $\sigma =0.05$, comprising 21 equispaced time points (including the initial condition). Another example with all three biological replicates, the same noise level, and 51 uniformly spaced measurements is presented in Figure 1C.

**Table 1:** Example parameter estimation values at different noise levels For given values of standard deviation measured in the Log-normal output distribution. Sect. 2.3 contains the details on the generation of synthetic data and a discussion of error magnitudes. Values are estimated using MLE, as described in Sect. 2.5.

$\sigma$	$\chi _{PB}^{\max }$	$\chi _{PB}^{\min }$	*a*	$\chi _{BP}$
Nominal	9.6	0.0960	0.0800	0.120
0.0025	9.722	0.1379	0.0771	0.132
0.0050	9.859	0.1801	0.0742	0.143
0.0100	10.15	0.2613	0.0686	0.166
0.0500	19.15	0.903	0.0214	0.359

We use MLE to estimate the parameters (see Sect. 2.5) from synthetic data. An example of the model with the best-fit parameters is displayed in Figure 1C. Qualitatively, we observe a good fit when visually comparing the curves in Figure 1C to the numerical solution in Figure 1B. Table 1 compares the recovered parameters for various noise levels up to $\sigma =0.05$ for 21 timepoint, 3 replicate, datasets. Despite visual agreement with the numerical solution, it is clear that the recovered parameter values sometimes differ from the nominal values. The estimation accuracy improves as the noise level decreases. Our ultimate goal is to understand whether, under the best data scenarios possible, we are able to accurately measure all parameters. Therefore, f or the remainder of this section, we will focus on the very-low noise case, $\sigma =2.5\times 10^{-3}$, since this case provides the best opportunity for parameter identifiability. This noise level also provides a significant, but measurable, confidence interval at some sampling levels for less identifiable parameters. We present analogous results for larger noise values in Supplementary Section 2.

We next turn to profile likelihoods to explore parameter identifiability based on equispaced sampling schedules. Figure 2 displays the univariate profile likelihood (see Sect. 2.5 for details) to determine confidence intervals for each parameter under 11 (blue dotted), 21 (red dashes), and 51 (green line) equispaced time points. $\bar{\ell }_p$ is $\chi ^2$ distributed, thus values of $\bar{\ell }_p$ greater than the threshold (displayed as a horizontal yellow line) all have a greater than 95% probability of being responsible for the synthetic dataset from which they are measured. Thus, the points where the blue, red, and green curves intersect this line represent the 95% confidence interval bounds of this estimation. We normalise using the nominal values on the *x*-axis in each plot to allow comparison of the relative widths between parameters. For all four parameters, the confidence intervals always contain the nominal value, and increasing the number of time points sampled consistently reduces the width of the confidence intervals, indicating improvement in measuring parameters. For the green curve (51 data points), the peak value and confidence intervals give a very tight interval around the nominal value for $\chi _{PB}^{max}$, Figure 2A. Parameters *a* and $\chi _{BP}$, displayed in Figure 2C and Figure 2D, respectively, are relatively close ($\pm \sim 10\%$) to the nominal value. The range of values on the x-axis is extended for $\chi _{PB}^{min}$ (Figure 2B) to allow the confidence interval of the green curves to be measured (white and grey regions are given for visual reference to compare to the other plot’s ranges). The sparser datasets (11 and 21 measurements) in Figure 2A and Figure 2C produce reasonable estimated value measurements, with only Figure 2A giving a measurable interval in the range examined in 11 measurement datasets. In Figure 2B, the sparser datasets do not generate confidence intervals despite the larger domain, while the dense dataset can eventually give a value on a much larger range. Further plots are presented in Supplementary Section 3 showing the profile likelihoods at 1% and 5% error.

**Fig. 3:**
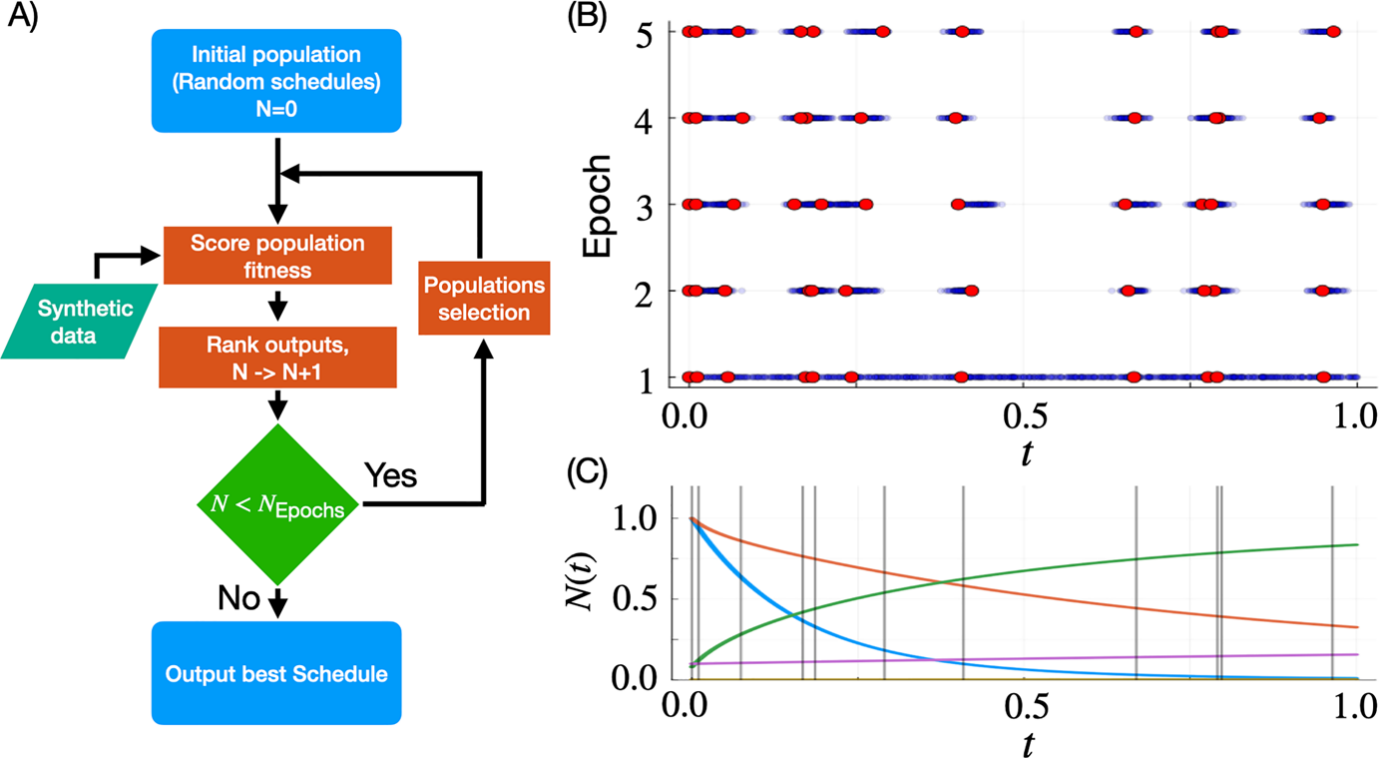
**Genetic algorithm schematic and examples.** (A) A flowchart highlighting the key steps of the genetic algorithm. Initially, 100 random schedules for the corresponding time points (11, 21, or 51) are fed in. For each, a synthetic dataset with three biological replicates is generated according to the schedule, and the $95\%$ confidence interval width is found for the parameter of interest. The schedules are then ranked by width, with the smallest widths ranking more highly. The process is repeated until the desired number of epochs has passed, with the best schedules being cloned between epochs (see section Section 2.6). Finally, the best schedule after the desired number of epochs is output. (B) An example of sample schedule evolution through multiple epochs is shown. The schedules are displayed as a low-opacity scatter plot for each epoch, with darker points indicating those that appear in multiple schedules. The best schedule within the epoch is shown as a set of larger red points. As the epochs pass (i.e., moving up the lines on the plot), these points move diffusively as they are cloned. (C) A plot of the dynamics in the system is shown. Here, *N*(*t*) is the population size of each compartment in the model at time *t*. For the best schedule (i.e., epoch 5 in (B)), the vertical lines shown represent the times at which sampling is optimal (color figure online)

### Applying Genetic Algorithms to produce optimal sampling schedules

**Table 2:** **Parameter confidence intervals for uniform and optimal sampling strategies and levels of data.** The 95% confidence interval widths are given for each parameter estimation. The columns titled U-N apply a uniform sampling schedule consisting of *N* sample points, including the initial condition. The Op-N columns have the genetic algorithm-optimised sampling schedules for *N* sample points, including the initial condition. Where no value could be estimated on the interval, the value NaN is given. The search interval was extended (see Figure 2B, where the grey region indicates the extended range) to quantify a value in the following best-case scenario where possible (e.g., 51 sampling times for $\chi _{PB}^{\min }$). The values are also visualised in Figure 4.

Parameter	U-11	U-21	U-51	Op-11	Op-21	Op-51
$\chi _{PB}^{\max }$	0.159	0.073	0.028	0.0177	0.0123	0.0107
$\chi _{PB}^{\min }$	NaN	NaN	NaN	NaN	NaN	NaN
*a*	0.480	0.188	0.071	0.0901	0.0708	0.0473
$\chi _{BP}$	NaN	0.417	0.216	0.500	0.373	0.216

Our previous results indicate that dense sampling is needed to identify parameters accurately when using uniform sampling. We now investigate whether alternative sampling schedules can improve parameter identifiability. We use a genetic algorithm to systematically evaluate numerous schedules and determine their corresponding confidence interval widths (see Sect. 2.6). This approach is compared with classical optimal experimental design in Supplementary Section 4. A flowchart of the algorithm, along with examples, is presented in Figure 3. Keeping the same total number of data points as in Figure 2, a corresponding optimal schedule was produced, using three replicates in each synthetic dataset, based on its ability to minimise the corresponding parameter’s 95% confidence interval.

**Fig. 4:**
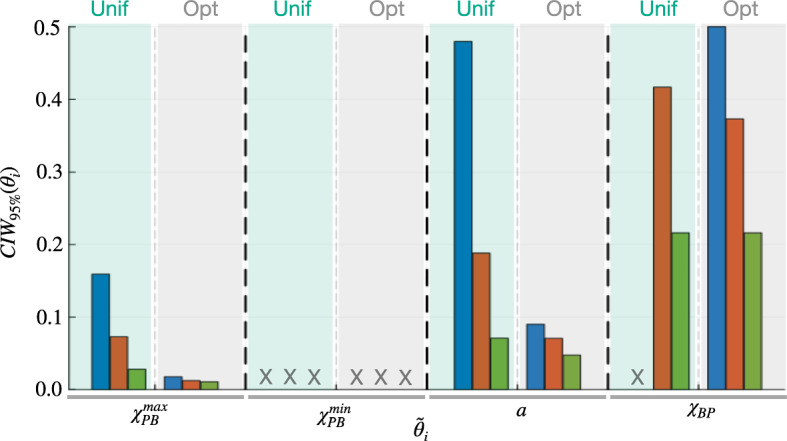
$95\%$ **confidence interval width for different sampling schedules and number of time-points.** Each parameter of interest has two sets of values, separated by light grey dashed lines, with parameters separated by dark black dashed vertical lines. For a given parameter, uniform time-point sampling values are given (light green columns), and optimal sampling from the genetic algorithm (light grey columns). The level of data with sampling types is 11, 21, and 51 time points, with blue, red, and green bars, respectively. Where no value could be found (i.e., $CIW_{95\%}>0.7$), a grey X is given instead of a bar (color figure online)

We compare the relative confidence interval widths between uniform and optimal sampling in Figure 4 and Table 2. For each parameter (separated by black dashed lines), there is a section representing uniform sampling (green background) and optimal sampling (grey background). Within each section, blue bars represent 11 time points, orange bars represent 21 time points, and green bars represent 51 time points. X’s represent a confidence interval larger than 0.7. As with uniform schedules, an increase in the available data (e.g., the number of time points) reduces the confidence interval, resulting in a more accurate parameter estimate. The trend in identifiability of the parameters is similar to uniform sampling, with $\chi _{PB}^{\max }$ and *a* showing the smallest interval widths, followed by $\chi _{BP}$, and then $\chi _{PB}^{\min }$, which was not identifiable under any circumstances. By optimally choosing our sample schedules, we achieve a confidence interval width for 11 optimally spaced measurements that is comparable to, and in some cases narrower than, that of 51 uniformly spaced measurements. However, there are diminishing returns for optimal sampling compared to the improvement seen in the uniform sampling case. For example, when comparing $\chi _{PB}^{\max }$ measured with 11 vs 51 sampling points, the confidence interval for uniform and optimal sampling was roughly one-sixth and one-half as wide, respectively. Even with optimal sampling, $\chi _{PB}^{\min }$ was highly non-identifiable, even in the highest data availability scenarios.

We observe stark differences in the identifiability of the parameters given these data scenarios. Overall, the maximal speed of binding $\chi _{PB}^{\max }$ is most readily measured and has the lowest associated uncertainty in all scenarios, suggesting that its value is essential in the overall dynamics of the system. The transition between fast $\chi _{PB}^{\max }$ and slow $\chi _{PB}^{\min }$ binding, governed by the value of *a*, as well as the binding off rate $\chi _{BP}$, were the following most readily measured parameters. This suggests they have some impact on the overall dynamics, but a lesser one than the maximal binding rate. Finally, we saw that the slow binding rate $\chi _{PB}^{\min }$ was not practically identifiable in all of the data scenarios. In contrast to $\chi _{PB}^{\max }$, the specific value didn’t readily impact the overall likelihoods, likely because the values were all small. As such, very few bacteria bind at this low rate, and so moderate changes to this tiny population are not easy to distinguish in light of the faster modifications dictated by the other parameters.

### Characterising Biofilm Size Sensitivity to Formation Parameters

**Fig. 5:**
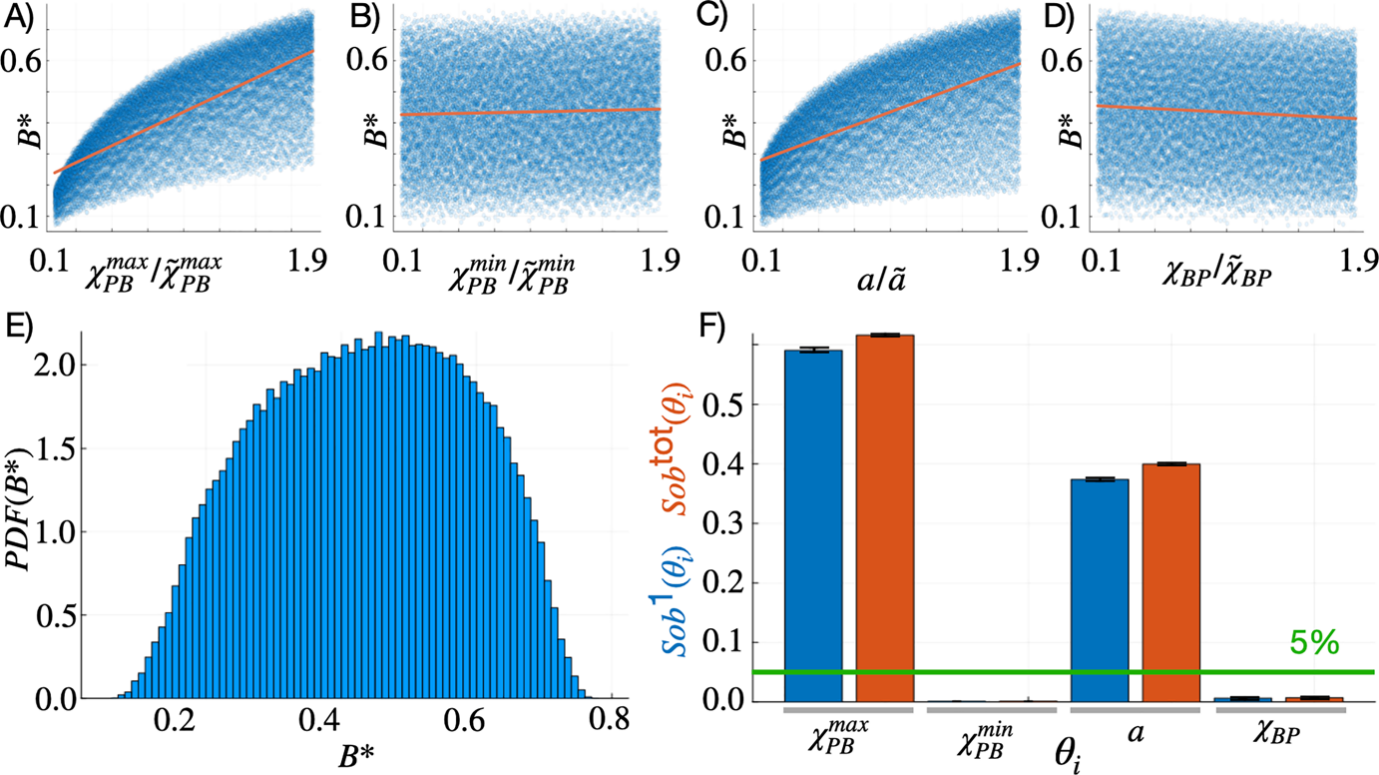
**Sensitivity analysis of the end-point biofilm size given variations in binding dynamics.** (A-D) Scatter plots of final biofilm size for parameters $\chi _{PB}^{max}$ (A), $\chi _{PB}^{min}$, (B), *a* (C), and $\chi _{BP}$ (D) over 40,000 Saltelli sampling parameter sets. For each scatter plot, a linear fit (red solid line) illustrates general trends in the data, and corresponding fit and correlation values are provided in Table 3. (E) The distribution of the end-point biofilm sizes across the parameter space, sampled from (A-D), is given. (F) The variance presented in (E) is decomposed to give the associated first-order (blue) and total-order (orange) Sobol sensitivity indices. Bars above the green horizontal line, representing the 5% threshold, indicate parameters that impact overall biofilm size (color figure online)

Having examined the identifiability of the different parameters, we next turn towards sensitivity analysis to determine the impact of varying these parameters on the final size of the biofilm, denoted $B^*$ (see details in Section 2.7). Figure 5A-D displays the univariate dependence of $B^*$ on the normalised underlying parameter. The best-fit line is displayed on these figures to highlight the general trend. Although the data are clearly non-linear by comparison to the fit line, this disparity motivates the use of Sobol Indices. Parameters are normalised to allow for a comparison of how proportional changes affect the output.

**Table 3:** Various sensitivity indices quantifying impacts on $B^*$ for select phenotype transition parameters. The table shows several metrics to quantify the dependence of the final biofilm size, $B^*$, on the model’s parameters.

Index type	$\chi _{PB}^{\max }$	$\chi _{PB}^{\min }$	*a*	$\chi _{BP}$
Intercept	0.218	0.426	0.263	0.460
Gradient	0.218	0.010	0.172	-0.023
Pearson	0.749	0.034	0.592	-0.078
Partial	0.935	0.118	0.901	-0.266
Spearman CC	0.014	0.005	-0.002	-0.001
PRCC	0.014	0.010	0.001	0.000
Sobol tot	0.616	0.001	0.400	0.007
Sobol 1st	0.590	0.001	0.374	0.006

Table 3 depicts several sensitivity indices of the univariate relationships captured in Figure 5A-D. The strongest linear relationships, measured by either the gradient of the linear fit (red lines Figure 5A-D) or the Pearson correlation coefficient, are present in $\chi _{PB}^{\max }$ and *a*. The remaining parameters only have weak linear dependence according to these metrics. The Partial Correlation for $\chi _{PB}^{\max }$ and *a* reinforces our observation, providing a clearer relationship when controlling for the other parameters. Interestingly, the magnitude of the Partial Correlation for $\chi _{PB}^{\min }$ and $\chi _{BP}$ increases in both cases, suggesting the influence of these parameters is being suppressed by variation in other parameters. In all cases, the Spearman and Partial Rank correlations (PRCC) are small. While linear metrics suggest a clear relationship, the near-zero rank-based indices (Spearman and PRCC) reveal that this signal is not robust to rank transformation. This indicates that the perceived correlation is primarily driven by the ‘pull’ of points at the extremes of the distribution (outliers); once the data is mapped to a uniform rank distribution, the high local density and variance in the mid-range effectively nullify the correlation signal. In Figure 5E, we can see this distribution of values, with the bulk of the values spread across $B^*=(0.2,0.8)$. The mean across the whole sample is $E(B^*)=0.435$, which is not far from the nominal value $B^*_0 = 0.484$. Finally, to decompose the relative impact of the parameters on $B^*$ regardless of their underlying relationship, we calculated the Sobol indices via a variance-based decomposition (Figure 5F). Supporting what has been seen up to this point, $\chi _{PB}^{\max }$ and *a* are dominant, with the sum of their first-order Sobol indices accounting for 95% of the total variance. Additionally, we can see that their total-order Sobol index is very close to their first-order Sobol index. Thus, the influence of these two parameters has an independent impact on the quantity of interest.

Following our concluding remarks from Sect. 3.2, the results seen here further suggest that the overall dynamics of the bacteria are strongly impacted by the value of $\chi _{PB}^{\max }$. That is, with planktonic bacteria quickly able to bind to biofilms, the result is a significantly larger difference in final biofilm size $B^*$. The value of *a* also has a relatively strong impact on this final biofilm size, likely since this value sets the duration for which the $\chi _{PB}^{\max }$ rate dictates the planktonic bacteria binding. As such, we see that bacteria that can maintain their fast-binding dynamics result in far larger final sizes $B^*$. The values of $\chi _{PB}^{\min }$ and $\chi _{BP}$ have relatively little impact on the final biofilm size, likely because their values are small. Therefore, bacteria that detach more readily or attach to large biofilms (i.e., $B>> a$) have relatively little impact on the overall final size of the biofilm.

### Capturing Bacterial Evolution by Selection with Structured Models

**Fig. 6:**
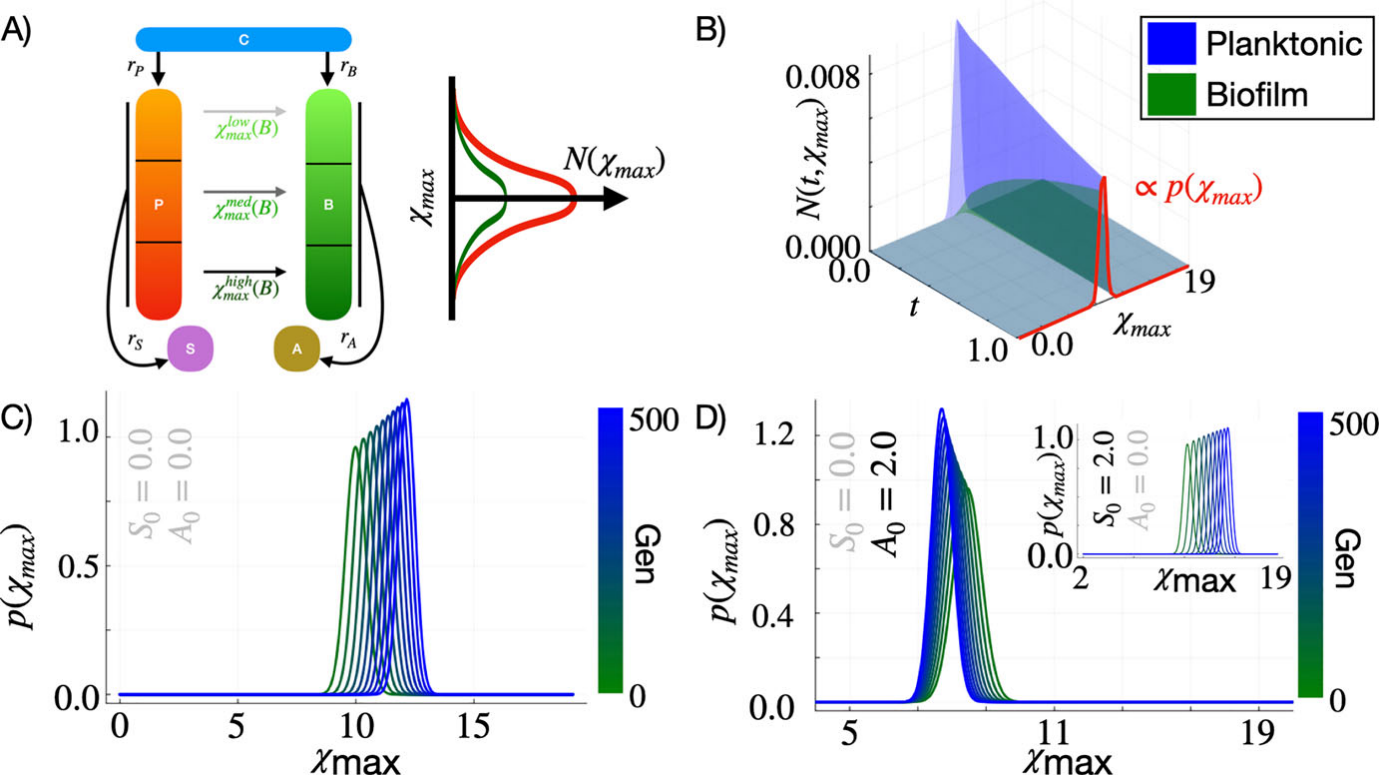
**Schematics, examples, and long-time dynamics of the phenotype parameter-structured model.** (A) A schematic of the parameter-structured population model (see Section 2.2). The key compartments are as in the short-term model: a media carbon source *C*, planktonic bacteria *P*, biofilm bacteria *B*, planktonic predator *S*, and biofilm predator *A*. Within the phenotype compartments, sub-communities are separated based on their ability to attach to the biofilm when in the planktonic phenotype. Within the populations *P* and *B*, the distribution of organisms with associated $\chi _{max}$ is initially normally distributed around some value $\bar{\chi }^i_{max}$. (B) An example evolution showing all of the compartments (here, characterised by differences in their $\chi _{max}$ value). The blue surface shows the time evolution of the planktonic compartments across one day, while the green surface shows the time evolution of the biofilm compartments. The height $N(t,\chi _{max})$ gives the population at time *t* within the population compartment $\chi _{max}$. The final time-point distribution of the biofilm’s $\chi _{max}$ values, $p(\chi _{max})$, is highlighted in red. (C) The final time-point distribution of $\chi _{max}$ within the biofilm is presented for 500 model generations. Ten curves are shown, given at time points uniformly spaced generations on 0 to 500. In this example, the model has no predation (i.e., $S_0$ and $A_0$ are zero throughout). The early curves are shown in green, while the later ones are given in blue. The curves can be seen moving slowly to the right as the generations increase. (D) The final time-point distribution of $\chi _{max}$ within the biofilm is presented for 500 model generations. Ten curves are shown, given at time points uniformly spaced generations on 0 to 500. The main figure models a population subject to a periodically reset population of biofilm predators. Here, the $\chi _{max}$ distribution drifts to the left. The inset models a population with a periodically reset planktonic predator. Here, the $\chi _{max}$ distribution drifts to the right (color figure online)

After evaluating the parameters that determine biofilm formation in the short term, we propose a structured model that includes a mechanism for long-term population changes through evolution (see Sect. 2.2). The key difference in our new model is that our system has multiple bacterial populations, each with distinct binding dynamics. An example schematic is presented in Figure 6A. This example features three compartments with different maximal binding rates, the values of which in the previous model were denoted $\chi _{PB}^{\max }$. Based on the results of the identifiability and sensitivity studies of the previous short-term model, we removed the parameters $\chi _{BP}$ and $\chi _{PB}^{\min }$, since their impact on the system was minimal. We note that the removal of these unidentifiable and insensitive parameters is dependent on both the initial conditions and bacteria-specific parameters. Additionally, instead of a scalar initial condition for *P* and *B*, the new populations $P_i$ and $B_i$ are given some initial distribution, namely normally distributed across some range of binding rates $\chi _{\max }$ values, with the *i*th compartment for some discretisation of this range having a value $\chi _i$.

As detailed in Section 2.2, we no longer focus only on simulations of single time intervals from $t=0$ to $t=1$, but instead take the state of the system at $t=1$ to reset the system periodically. In this way, it simulates multiple generations of the population subjected to a consistent and resetting environment. Figure 6B presents an example of the time interval $t=0$ to $t=1$ for these compartment populations. At $t=1$, our biofilm population is highlighted by a red curve; this population is used to reseed the planktonic population in the next generation.

We demonstrate the presence of selection by measuring the average binding rate $\bar{\chi }_{\max }$ present in our model both under no predation (Figure 6C) and with predation (Figure 6D). These figures display the probability distribution of the compartments $\chi _{\max }$ values. Without predators, the distribution shifts to the right, going from a non-dimensional mean $\bar{\chi }_{\max }$ of 9.6 to 12.2, representing a 27% increase, as the system undergoes 500 generations. This right shift indicates that bacteria with a higher $\chi _{\max }$ value (i.e., those that more readily attach to the biofilm) are more represented at the end of each generation and are therefore being reseeded into the next generation. Biologically, bacteria which rapidly form biofilms are better able to overcome the selection pressures posed by the experimental setup, since only bacteria in the biofilm move to the next generation. The increased peak height suggests that the distribution is narrowing over time, slowing the rate at which the curve moves to the right. This suggests that there is some kind of trade-off that is eventually leading to a slowing down of adaptation. As a result, bacteria which rapidly form biofilm eventually become less favoured, which is likely because reproduction (bacterial growth) is much slower when bacteria attach to a biofilm. Under no predation, we are naturally selecting for bacteria that can quickly attach to the biofilm, as these are the ones that make it to the next generation. The evolutionary effects of predator stress are displayed in Figure 6D. The main figure features biofilm predators, and the inset figure has planktonic predators, both of which are periodically refreshed. The biofilm predator drives opposite selection relative to the no-predator case, with the curve shifting to the left and the mean of $\chi _{\max }$ decreasing from 9.6 initially to 8.8, representing an 8% decrease, favouring smaller values of $\chi _{\max }$, where bacteria transition to the biofilm more slowly. This change looks relatively small. However, without predators, the mean reaches 12.2, an effect that biofilm predators can easily offset and reverse. Bacteria which form biofilms more slowly spend longer in the planktonic phenotype, where they are protected from biofilm-specific predators. As before, this curve is narrowing, indicating that the process slows down as the bacteria undergo more generations. The planktonic predator dynamics look similar to the no-predator case, with bacteria with a higher $\chi _{\max }$ being favoured; however, it shifts right at a faster rate, reaching a final mean of 14.4, representing a 50% increase. That is, bacteria which more readily form biofilm are favoured, since being in a biofilm isolates them from this selection pressure. The presence of predators results in very different types of selection within this model, which drives different outcomes in the bacterial population.

### Characterising Evolutionary Dynamics’ Sensitivity

**Fig. 7:**
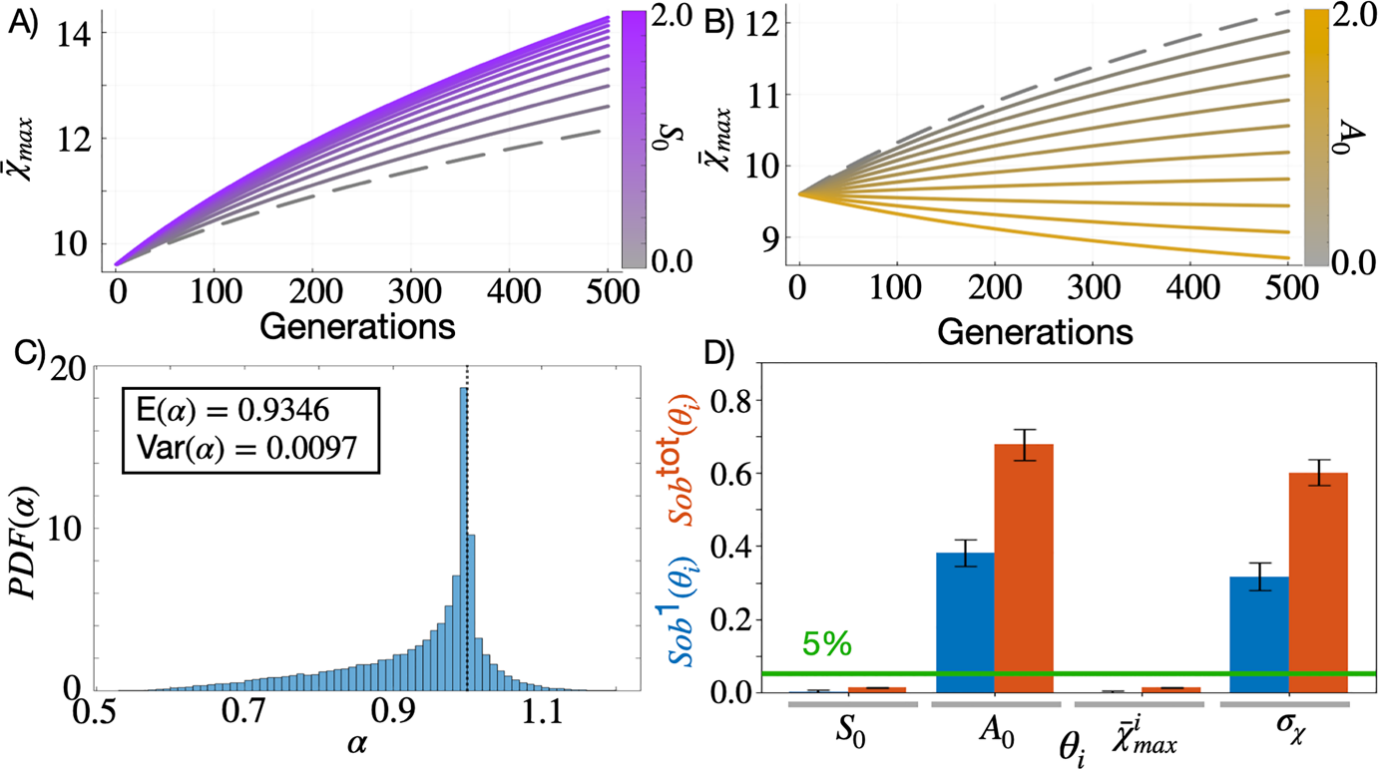
Characterising trends within the $\chi _{max }$ distributions across generations and measuring the sensitivity of these trends to conditions. The biofilm’s average binding rate, $\bar{\chi }_{max}$, for 10 varying amounts of resetting predator populations for planktonic (A) and biofilm (B) predators over 500 generations. The colour bar provides the size of the resetting predator population for each model. (C) The distribution of the quantity of interest $\alpha =\left( \frac{\bar{\chi }_{max}^f}{\bar{\chi }_{max}^i}\right)$ (i.e., the ratio of final to initial mean binding rate) at 50 generations. The inset gives the mean and the variance of the Saltelli samples. (D) The variance presented in (C) is decomposed to give the associated first-order (blue) and total-order (orange) Sobol sensitivity indices (color figure online)

To further understand the relationship between predation and bacterial populations, we can examine how the number of predators within the system affects their evolution. In Figure 7A and Figure 7B, we measure the average binding rate in the population, $\bar{\chi }_{\max }$, at the end of each generation. Figure 7A shows that over 500 generations, the population subjected to planktonic predators tends toward higher values of $\bar{\chi }_{\max }$, indicating that biofilms are forming more quickly. These predators compound the selection effects in the no-predator system, further increasing the value of $\bar{\chi }_{\max }$ in the population. In contrast, Figure 7B shows a stronger relationship between the predator presence and the binding rate. Interestingly, Figure 7B transitions from an increasing $\bar{\chi }_{\max }$ value to a decreasing one as the predator quantity increases, as the biofilm predators overcome the selection from the experimental design. All the curves in Figure 7A and Figure 7B show varying slowing levels, with the gradient over time decreasing, suggesting each predator level may drive some asymptotic binding value on very-long timescales.

To understand the most significant drivers in selection for biofilm formation, we undertook a sensitivity study on the structured model (described in Section 2.8). We examined the effects of (1) the reset population of the planktonic predator $S_0$, (2) the biofilm predator $A_0$, (3) the initial population mean binding rate $\bar{\chi }_{\max }^i$, and (4) the population initial standard deviation $\sigma _\chi$. The quantity of interest measured for our sampling was the ratio of the final population average binding rate to the initial. We used measurements at 50 generations based on the results presented in Figure 7A and B. These figures clearly distinguish their population’s average binding rates at 50 of the total 500 simulated generations. Since these simulations are relatively computationally expensive, this choice balances execution times with simulations that are long enough to allow a clear distinction to result from the selection impacts. We examined a sample across the parameter space using the Sobol sampling method. Figure 7C displays the observed values distribution. We can see that the distribution is skewed to the left, with most of the samples reducing the final average binding rate compared to the initial. After measuring the overall variance in this distribution, we calculated the Sobol sensitivity indices to determine which parameters drive these variances (Figure 7D). Most of the variation is attributed to the variance of the parameters $A_0$ and $\sigma _\chi$, the resetting biofilm predator population size, and the initial bacterial binding distribution variance, respectively. Unlike our previous sensitivity analysis, the first-order and total Sobol indices were unequal, indicating that the parameters are no longer independent. Instead, higher-order effects are also at play. The second-order indices have no significant effect, except the index between $A_0$ and $\sigma _\chi$, which had a value $0.274 \pm 0.055$. Given first-order index values of $A_0$ and $\sigma _\chi$ of 0.381 and 0.317, respectively, the combination of these first-order values and the measured second-order between them explains $97.2\%$ of the overall variance in the data. This suggests that initial populations with solely high parameter variance, or solely under high levels of predation, are insufficient to drive the most significant levels of change. Instead, these two factors cooperate, driving the most considerable deviations from the nominal.

These results suggest that bacterial populations subject to such biofilm predators should be expected to display signs of selection in their biofilm formation dynamics most rapidly (i.e., the resulting selection quickly selects for bacteria which form biofilms more slowly). While planktonic predation will drive some changes in biofilm formation dynamics, these changes occur more slowly, likely because of the cooperation between protection from predation, as well as being well placed to be re-seeded in the next generation, if the bacteria are able to make it into the biofilm. For less diverse populations of bacteria, as is common in lab strains, this lack of diversity likely limits the rates of selection since there is limited capacity of sub-populations to outperform others.

## Conclusions

This study investigated models of bacteria switching between planktonic and biofilm-forming phenotypes, with results presented in two main areas. First, we explored the short-term population dynamics of bacteria under predation stress, assessing the real-world applicability of a mathematical model through structural and practical identifiability, optimal design, and sensitivity analyses. Recognising the short-term model’s limitation in reflecting long-term population changes, we then proposed a new structured long-term model. Our minimal approach divided phenotypic populations into compartments with varying behaviours, allowing some to outperform others. By periodically resetting our system and preserving the population distribution of this transition behaviour, mirroring the Long-Term Evolution Experiment (LTEE) design often conducted in laboratory settings, we enabled selection to occur within our model.

In the first part of our study, we analysed an existing stoichiometric model that describes bacteria transitioning between planktonic and biofilm phenotypes and their associated predators. We first confirmed the system’s global structural identifiability when information from all state variables was available. Furthermore, we demonstrated that restricting the parameters to those involved in biofilm dynamics ($\chi _{PB}^{\max }$, a, $\chi _{PB}^{\min }$, and $\chi _{BP}$), only information about the biofilm state variable (*B*), is required for global structural identifiability.

We then generated synthetic datasets from numerical solutions to estimate these biofilm dynamics parameters. We used Maximum Likelihood Estimation to estimate parameters from synthetic data. We found that, while the fitted solutions accurately reproduced the overall trends in the data, our practical identifiability study revealed that the accuracy of parameter reconstruction varied with sampling frequency and scheduling. In a best-case scenario, across a low-noise scenario with 11 to 51 measurements, the maximal binding rate of planktonic cells ($\chi _{PB}^{\max }$) and the biofilm size at which the binding rate decreased (*a*) exhibited the narrowest confidence intervals. This was likely due to initial conditions featuring a large planktonic population and a small biofilm, as is common in experimental setups. Conversely, $\chi _{PB}^{\min }$ and $\chi _{BP}$ were more challenging to measure accurately, likely because of their limited activity periods and smaller magnitudes. We propose that modifying experimental protocols, such as pre-growing biofilms, could enhance the measurement of these less identifiable parameters by shifting initial conditions to emphasise their roles in the dynamics. However, this approach might compromise the accuracy of other parameter measurements. Finally, our application of a genetic algorithm revealed that optimised sampling schedules could achieve data quality comparable to, or even exceeding, that of significantly higher uniform sampling rates, suggesting a limited inherent informativeness of state variables regarding underlying parameters.

The sensitivity analysis on the initial model clearly demonstrated that $\chi _{PB}^{\max }$ and *a* dominated the system’s overall variance. These results, combined with our parameter identifiability findings, led us to simplify the model for initially small biofilms. Thus, in our long-term structured model (Section ()), we omitted the biofilm-to-planktonic transition rate ($\chi _{BP}$) and the large-biofilm binding rate ($\chi _{PB}^{\min }$). Our long-term model organises planktonic and biofilm populations into compartments, each defined by a unique maximum transition rate ($\chi _{\max }$) from planktonic to biofilm. Initially, $\chi _{PB}^{\max }$ values are normally distributed within the planktonic bacteria. While the short-term evolution of this system qualitatively resembles the initial model, periodic reseeding using the biofilm’s $\chi _{\max }$ distribution generates emergent differences over many generations. Selection was demonstrated to occur even in the absence of predators; this is likely driven by the reseeding mechanism, which acts as a selective pressure.

We observed that the presence of predators significantly alters the dynamics of selection, as has been hypothesised in the literature (Matz and Kjelleberg 2005). The biofilm-specific predator (*A*) had a significantly greater impact, resulting in faster adaptation. Specifically, exposure to grazing by biofilm predators reduced biofilm formation. In contrast, a planktonic predator (*S*) had a smaller effect compared to the inherent selection in the no-predator scenario. The effect was that the populations formed biofilms more quickly. Previous experimental work has demonstrated that grazing by ciliates (e.g., planktonic predators) can either inhibit (Huws et al. 2005) or stimulate biofilm formation (Kaminskaya et al. 2007). Subsequent work has argued that protozoan grazing initially inhibits biofilm formation, but later stimulates its growth (Rychert and Neu 2010). Interestingly, while the biofilm predator strongly influenced adaptation, the rate of adaptation slowed down more rapidly than in the other cases. This suggests a trade-off: organisms must transition quickly enough to form biofilm by the end of the generation, but not so quickly that they risk prolonged exposure and consumption within the biofilm. Consequently, the distribution of $\chi _{\max }$ shifts towards an optimal value that balances this trade-off.

We performed sensitivity analysis to understand how the ratio between final and initial mean binding rates is impacted by key model inputs: initial planktonic predator population size ($S_0$), initial biofilm predator population size ($A_0$), initial bacterial mean binding rate ($\bar{\chi }_{\max }$), and initial population distribution variance ($\sigma _\chi$). This analysis revealed that variance is dominated by biofilm predator ($A_0$) and initial population variance ($\sigma _\chi$). The interaction between $A_0$ and $\sigma _\chi$ accounted for most of the unexplained variance. This is because $A_0$ directly impacts the biofilm population, exerting a strong and clear selective pressure. Similarly, a larger $\sigma _\chi$ enhances the system’s capacity to respond rapidly to selection, as it provides a greater initial diversity of competitively advantageous populations.

Our current models offer several avenues for future exploration. Although a comprehensive investigation of genetic algorithms was beyond the scope of this study, we found that optimal sample points often clustered. Future research could address this by either removing these clustered points or replacing them with new, randomly drawn points that maintain a minimum pairwise distance. Additionally, minimising the sum of all confidence intervals, as suggested by (Lam et al. 2024), could yield more robust sampling schedules. Finally, data sets incorporating multiple initial conditions could be employed to yield more accurate parameter set measurements, especially in models with regime-dependent identifiability.

Our structured model effectively captures selection dynamics; however, there is a need for further refinement to reflect real-world complexities, particularly the inherent limitations in bacterial cellular processes. Currently, our model simplifies these by characterising compartment differences solely based on $\chi _{\max }$ values, which could represent variations in attachment ability. However, selection likely also involves other parameters, such as bacterial reproduction rates ($r_P$ and $r_B$). Furthermore, real biological systems may impose discrete or bounded constraints on $\chi _{\max }$ values, and multiple underlying parameters often vary simultaneously under stress, potentially incurring energetic costs for traits like increased $\chi _{\max }$. Our model also assumed that all possible $\chi _{\max }$ values were initially present. To create more realistic models of systems, future studies should explore constrained and simultaneous parameter changes, incorporate mutation dynamics between compartments, and crucially, align these model additions with experimental observations to avoid unjustified theoretical assumptions.

## Supplementary Information

Below is the link to the electronic supplementary material.

Supplementary file 1 (pdf 734 KB)

## Data Availability

All data generated and codes used in analysis during this study are included in the published article’s associated GitHub at https://github.com/StephWilleniams/BiofilmModelAnalysis.

## Declarations

### Code availability

The code is available on GitHub at: https://github.com/StephWilleniams/BiofilmModelAnalysis. Where possible, fixed random seeding was used for all synthetic data in the figures to ensure reproducibility of all the data. Where practical, Julia was used to execute the calculations. The ModelAnalysis module was developed during the work and provides much of the functionality used for practical identifiability and genetic algorithm development. For convenience, some tasks required execution using MATLAB or Python. These tasks utilised the simple vectorisation and parallelisation capabilities that MATLAB allows.
