# Supplementary material for: Identifiability, Sensitivity, and Genetic Algorithms in Bacterial Biofilm Selection Models
Source: Bull Math Biol. 2026 Jul 2;88(7):132. doi: 10.1007/s11538-026-01693-5 (PMC13328132; doi:10.1007/s11538-026-01693-5)
Supplement: Supplementary file 1 — (pdf 734 KB) [file 11538_2026_1693_MOESM1_ESM.pdf]

Supplementary Materials: Identifiability, Sensitivity, and Genetic Algorithms in Bacterial Biofilm Selection Models

Stephen Williams[1], Daravuth Cheam[2,3], Michele K. Nishiguchi[2], Suzanne S. Sindi[1], Shilpa Khatri[1], and Erica M. Rutter[1*]
[1]University of California, Merced, Department of Applied Mathematics
[2]University of California, Merced, Department of Molecular and Cell Biology
[3]University of California, Merced, Quantitative Systems Biology Graduate Group
[*]Corresponding Author: erutter2@ucmerced.edu

1 Nondimensionalisation of the Model

Variable	Name	ND Value	Value	Units	Conversion Factor
	Fixed parameters				
e_b	Bacterial growth efficiency	0.2			
r_P	Planktonic growth rate	25.2	0.21	h^{-1}	T_{ND}/e_b
H_P	Planktonic Half-Saturation	3.0	1	μgC ml^{-1}	V
r_B	Biofilm growth rate	0.84	0.007	h^{-1}	T_{ND}/e_b
H_B	Biofilm Half-Saturation	3.0	1.0	μgC ml^{-1}	V
g_S	Planktonic predator growth rate	5.76	0.12	μgC ml^{-1}	T_{ND}/e_S
e_S	Planktonic predator growth efficiency	0.5			
H_S	Planktonic predator half-saturation	3.0	1.0	μgC ml^{-1}	V
g_A	Biofilm predator growth rate	4.32	0.09	h^{-1}	T_{ND}/e_A
e_A	Biofilm predator growth efficiency	0.33			
H_A	Biofilm predator half-saturation	0.8	0.1	μgC cm^{-2}	A
	Re-estimated parameters				
a	Attachment strength parameter	0.08	0.01	μgC cm^{-2}	A
$\chi_{PB}^{\max}$	Biofilm attachment rate maximum	9.6	0.05	cm h^{-1}	$T_{ND} * A$
$\chi_{PB}^{\min}$	Biofilm attachment rate minimum	0.096	0.0005	cm h^{-1}	$T_{ND} * A$
χ_{BP}	Biofilm detachment rate	0.12	0.005	h^{-1}	T_{ND}
	Initial conditions				
$C(0)$	Initial media concentration	1.0	0.33	μgC ml^{-1}	V
$P(0)$	Initial plankton concentration	1.0	0.33	μgC ml^{-1}	V
$B(0)$	Initial biofilm concentration	0.01	0.00125	μgC cm^{-2}	A
$S(0)$	Initial ciliate concentration	0.03	0.01	μgC ml^{-1}	V
$A(0)$	Initial amoeba concentration	0.008	0.001	μgC cm^{-2}	A

Supplementary Table 1: **Population model parameters compared to original values** Table of each of the parameters present in Equations (1)-(5), and Main Figure 1A. The values are a nondimensionalization of those presented in (Seiler et al., 2017); see Section 1 for details on this process. A brief description of each nominal non-dimensional value is given. The parameter values, or our dimensionalised value equivalents, from (Seiler et al., 2017), and their units and conversion factors are given in the final two columns, where T_{ND}, the time used to non-dimensionalise the parameters, is 24 hours. The first set of parameters is fixed and well-known within our model throughout. The second set (a, $\chi_{PB}^{\max}$, $\chi_{PB}^{\min}$, χ_{BP}) are the unknown parameters, which we consider the variations of. Their nominal values used to generate synthetic data are given. The final set of five values then provides the initial condition values used for simulations, unless otherwise specified.

In the original model, as presented in (Seiler et al., 2017), the carbon, planktonic, and planktonic predators have units of carbon per volume. The biofilm and its associated predator have units of carbon per area. These units are generally convenient, giving the closest description to what would be measured experimentally. However, the mixed-compartment units in the model can provide a somewhat obscured comparison of the relative sizes of the populations, making them challenging to compare. As such, the equations describing the populations were multiplied by their associated dimensions to avoid inconsistent comparisons, either a volume of 3ml or an area of 8cm^2. With this change, the populations represent the total quantity of carbon stored in each state variable. For consistency, several parameters must also be handled this way, for example, H_C. Upon this dimensionalisation, the area/volume value in the original model equations was absorbed into other parameter values, where necessary. As such, the values of $\chi_{PB}^{\max}$ and $\chi_{PB}^{\min}$ have been scaled by the original system's area, and a by the system area. In addition, the parameters and timescales were also normalised so that 1 time unit corresponds to 1 day (24 hr), since we entirely focus on simulated runs of this length. The efficiency calculation is performed to enforce model structure consistency, and the values of r_p and r_b are scaled by $1/e_b$. The efficiency value e_b is applied in Equations (2) and (3), to be consistent with how it is used for Equations (4) and (5). Supplementary Table 1 depicts the original population model parameters and our population model parameters.

We choose our initial conditions for the system to be $C = 1$ and $P = 1$, corresponding to $0.3\dot{3}\mu$g/ml in the dimensionalised units; the remaining populations are then chosen to be some orders of magnitude smaller. This choice provides a convenient way to consider how these populations evolve relative to their initial condition, which becomes an essential consideration in Section 2.2 to facilitate a simple reseeding process.

A limitation of this nondimensionalization approach is that the system's parameter values are then tailored to the specific geometry, since the underlying parameters in (Seiler et al., 2017) were scaled to fit the given experimental container. Another potential approach was scaling the state variables and parameters to the initial carbon content sum across compartments. This normalisation would allow a convenient way to view how the carbon is distributed proportionally. Additionally, that would clarify what carbon is lost through efficiency (e_b, e_S, e_A). However, the initial conditions favouring scaling were implemented, since we are primarily interested in between-parameter comparisons.

2 Higher noise parameter identifiability

We recalculated the results presented in Main Figure 2 for each parameter to compare the confidence intervals with higher-noise cases. Supplementary Figure 1 displays the profile likelihoods for 1% error (top) and 5% error (bottom) for all four parameters. In these cases, the 95% confidence interval widths we observe follow a similar trend to that in the low-noise dataset presented in the paper. Throughout, each 95% interval correctly contains the nominal value. Here, $\chi_{PB}^{\max}$ and a show the tightest intervals, while $\chi_{PB}^{\min}$ and χ_{BP} are practically non-identifiable at this level of noise.

Additionally, we repeated the genetic algorithm approach for these higher noise levels, when applied to an 11-time point schedule. Supplementary Table 2 presents the bounds for each parameter under 1% and 5% noise. We observe that, similar to the low-noise case presented in the main text, $\chi_{PB}^{\min}$ and χ_{BP} remain non-identifiable under optimal scheduling. $\chi_{PB}^{\max}$ and a are more identifiable, although a becomes non-identifiable at the highest noise values.

σ	$\chi_{PB}^{\max}$	$\chi_{PB}^{\min}$	a	χ_{BP}
1%	0.0595	NaN	0.3159	NaN
5%	0.351	NaN	NaN	NaN

Supplementary Table 2: **Genetic algorithm optimised schedule bound estimates.** The 95% confidence interval widths for the parameter, calculated using genetic-algorithm-optimised schedules, are presented. The top row shows synthetic data with a 1% noise level, and the bottom row shows 5%. Where it was not possible to estimate a 95% confidence interval (i.e., bound widths greater than 100% of the nominal value), NaN has been inserted.

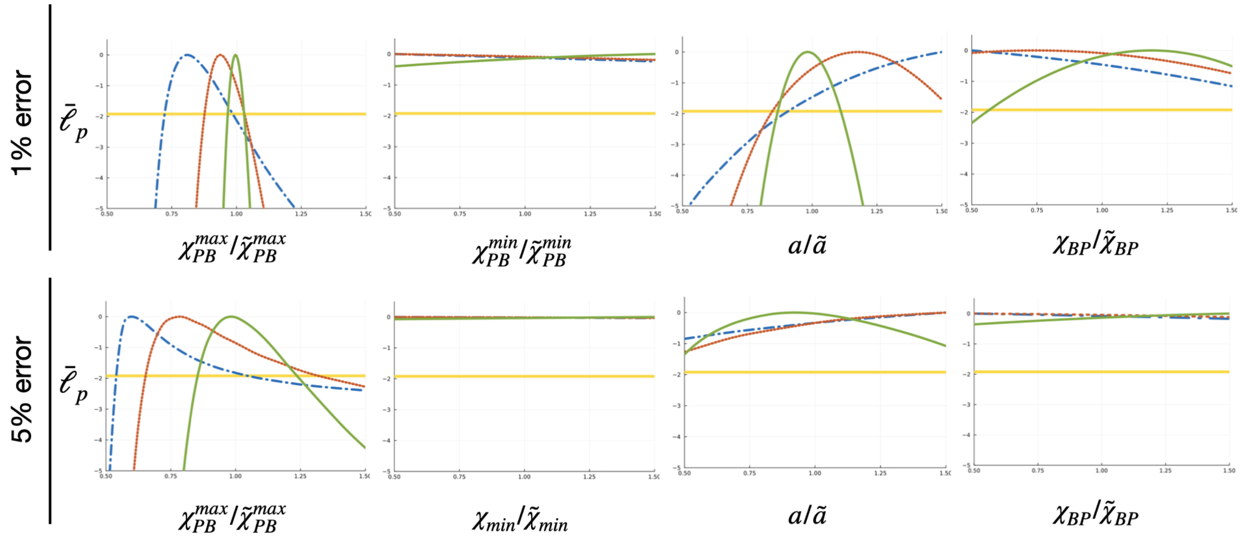

Supplementary Figure 1: **Higher noise-level profile likelihoods.** The profile likelihood for each of the four biofilm parameters is shown. The top row shows synthetic data using a 1% mean error. The bottom row shows synthetic data using a 5% mean error. Each plot has the 95% confidence shown as a yellow horizontal line.

3 Multiple start optimisation

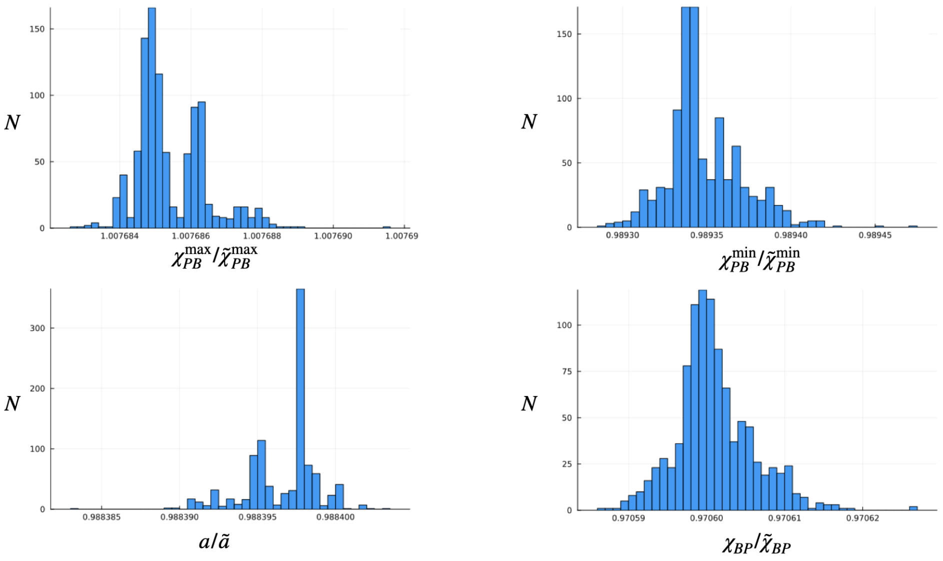

Supplementary Figure 2: **Histograms of multiple initial guess parameter estimates.** Histograms of the final estimated parameter values are shown for 1000 randomly chosen initial parameter guesses in the optimisation. The four panels correspond to the four parameters varied in the main results. In each case, the x-axis is normalised to the nominal value to show the difference relative to it.

To confirm that our results do not depend on the initial guess used in the optimiser, we performed 1000 runs using Sobol sampling. The initial guesses used in this trial were within ±20% of the nominal values. Using these randomly sampled initial guesses, we then re-estimate the parameters for an identical set of synthetic data. The synthetic data uses $\sigma = 0.005$, 51 time points, so it's expected to be very accurate. However, the noise is set to a higher value than the $\sigma = 2.5 \times 10^{-3}$ used in our main results, to provide a worst-case-scenario estimate.

We can see the results of this multiple-start optimisation in Figure 2. The results result in a very tight range of estimated values. For $\chi_{PB}^{\max}$, all output values are the same up to 1.00768 ± 0.000005, for $\chi_{PB}^{\min}$ outputs are 0.989± 0.0005, for a outputs are 0.988 ± 0.0005, and . for χ_{BP} outputs are 0.970 ± 0.0005. For each parameter, the variance across the random samples is negligible. By comparing these to the bounds of our 95% confidence intervals for each parameter (see Fig. 2 in the main text), all measured values are well within the 95% confidence intervals obtained across 51 time points. Additionally, comparing the initial guess induced variance to the size of the bounds, it is not expected to affect any of our results, even estimated at this higher noise level in our synthetic data. Based on these results, we opt to use a single start for the remainder of the results, as many of the calculations (i.e., identifiability and sensitivity) are computationally expensive, which makes wide initial guess sampling prohibitive.

4 Optimal experimental design comparison

To compare the results of the genetic algorithm with traditional optimal experimental design (OED) techniques, we generated D-optimal schedules. To do this, we determined the optimal time schedule using the nominal parameters to maximise the possible improvements of parameter estimation quality. For an initially random set of time-points, we generate a synthetic dataset, and we can then measure the log-likelihood, $L(\boldsymbol{\theta}|\boldsymbol{Y})$, of this data given the nominal parameters (see Main Section 2.5).

From this likelihood (Qi and Baker, 2025), we can calculate the Fisher Information Matrix

$$F_{ij} = -E\left(\frac{\partial^2}{\partial \log(\theta_i)\partial \log(\theta_j)} L(\boldsymbol{\theta}|\boldsymbol{Y})\right). \quad (1)$$

where the expectation is calculated across the dataset.

For a D-optimal experimental design, we then optimise the schedule times to maximise $\log(\det(\boldsymbol{F}))$, thereby maximising the information content of the measurements.

Convergence in schedule space depends on the initial schedule, so we use 15 independent start schedules to overcome this. For these, we then choose the schedule which maximises the information content. Additionally, we add a quadratic penalty term to the points in the schedule, $\log(\det(\boldsymbol{F})) + \text{penalty}$, where

$$\text{penalty} = \sum_{i<j} \kappa H(t_c - |t_i - t_j|)(t_c - t_i)^2. \quad (2)$$

The value of $\kappa = 10^8$ provides a strong repulsion between the points, H is a Heaviside step function, and $t_c = 0.1$ is a critical distance below which the points are subject to the penalty. In the penalty-free optimisation, unlike the genetic algorithm approach, where points are sometimes paired up, points are highly clustered. The OED clusters primarily at the start and end of the time range. Such schedules are often impractical to implement in practice, so the inclusion of this penalty makes them more reasonable.

Supplementary Table 3 displays the results of the baseline (uniform) sampling, genetic algorithm, and our two optimal experimental design approaches outlined above. The unconstrained and genetic algorithm approaches produced comparable results for the 95% confidence interval. The constrained algorithm is an improvement over the baseline but fails to achieve bounds comparable to the unconstrained and genetic algorithm approaches. The OED approach, despite being conceptually more complex, is both more established in the literature and substantially faster computationally. In all optimisation approaches, further work remains to understand point clustering.

To examine whether the optimized schedules were similar, we plotted the resulting data collection schedules for the parameter $\chi_{PB}^{\max}$ at a noise level of 0.25% in Supplementary Figure 3. Despite the genetic algorithm and unconstrained optimal experimental design having similar confidence interval widths, the unconstrained optimal experimental design schedule is very clustered.

parameter	σ	baseline	GA	OED	OED + penalty
$\chi_{PB}^{\max}$	0.25%	0.159	0.0177	0.0133	0.0623
$\chi_{PB}^{\max}$	1%	0.738	0.0568	0.0595	0.133
$\chi_{PB}^{\max}$	5%	NaN	0.400	0.351	NaN
$\chi_{PB}^{\min}$	0.25%	NaN	NaN	NaN	NaN
$\chi_{PB}^{\min}$	1%	NaN	NaN	NaN	NaN
$\chi_{PB}^{\min}$	5%	NaN	NaN	NaN	NaN
a	0.25%	0.48	0.0680	0.0901	0.207
a	1%	NaN	0.357	0.3159	0.468
a	5%	NaN	NaN	NaN	NaN
χ_{BP}	0.25%	NaN	0.432	0.500	NaN
χ_{BP}	1%	NaN	NaN	NaN	NaN
χ_{BP}	5%	NaN	NaN	NaN	NaN

Supplementary Table 3: **Optimal experimental design parameter 95% confidence intervals.** The 95% confidence interval for the parameter under a D-optimal experimental sampling schedule is given. Schedules consist of 11 time points. Values are given at various noise levels, σ. The baseline value present in the table was measured by uniformly spaced sampling times. The genetic algorithm approach values are shown in the GA column Both non-constrained and constrained schedules are present. NaN is given where no interval could be measured.

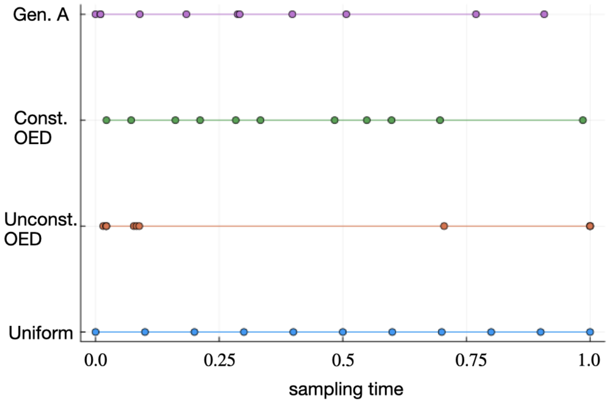

Supplementary Figure 3: **Optimized Data Collection Schedule Comparison.** Uniform, genetic algorithm, and both optimal experimental design schedules plotted for 0.25% noise and minimizing the 95% confidence interval for $\chi_{PB}^{\max}$.

5 Compartment convergence in the long-term model

To assess the convergence properties of our long-term model with respect to the number of compartments, we conducted a basic convergence study.

Here, we solved Equations (6)-(12) at t=1 after 500 generations. In this analysis, we calculate the final planktonic population $F_i^{N_c}(1;500)$ under identical conditions, varying the number of bacterial compartments N_c from $N_c = (11, 21, 41, ..., 2561)$. From the solution, we compare each N_c-case compartment with the coarsest case, $N_c = 11$, to assess point-wise convergence. We define the norms for this as

$$L_2^j = \sqrt{\frac{1}{11}\sum_{i=1}^{11}\left(F_i^{N_c^j}(1) - F_i^{N_c^{j+1}}(1)\right)^2}. \tag{3}$$

$$\tag{4}$$

Modelling the error as a function of compartment size (i.e., $\rho = 1/N_c$, we expect a convergence in compartment size of order β following the relationship

$$L_2(\rho) = \alpha\rho^\beta + o(\beta + 1). \tag{5}$$

In Supplementary Figure 4, we can see a plot of the 2 norm plotted in this way. In the limit of a few sampling points, the convergence is approximately of order 13 (i.e., $\beta = 13$; order 10 is shown in the figure for reference) and quickly settles to machine precision, with larger compartment counts reducing the measured point-wise error. In our long-term model simulations, the discretisation error reaches machine precision (the $N_c = 641$ case is indicated). This occurs because the compartments, while representing a continuum of trait values, do not interact directly; they are coupled only globally through the total biofilm (B_{tot}) and planktonic (P_{tot}) populations. Consequently, the individual rates of change within the ODE system can be evaluated without spatial approximation. The only numerical approximation arises when computing B_{tot} and P_{tot} as sums. Because our initial trait distribution is smooth and bounded, the integral converges exponentially, ensuring that our chosen grid accurately captures continuum dynamics without introducing truncation error. In our results, we use $N_c = 2000$ to ensure that the simulations clearly exceed spectral convergence, and the resulting computational time is reasonable for sensitivity analysis (which requires many runs).

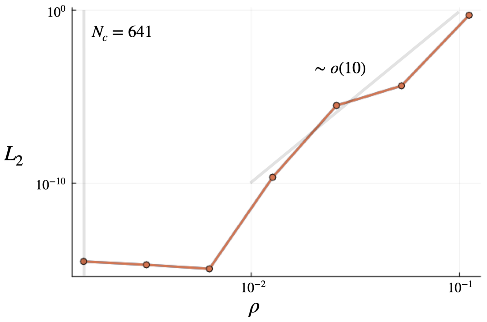

Supplementary Figure 4: **Convergence plot of the long-term model.** The 2-norm of the model's point-wise error is shown. A sample gradient is provided for order-10 convergence. The number of compartments N_c for the largest case is also shown.
